# Reliability of the PREFIT fitness-test battery in Chilean preschoolers

**DOI:** 10.3389/fped.2025.1654731

**Published:** 2026-01-12

**Authors:** Johana Soto-Sánchez, Barbara Leyton-Dinamarca, Juliana Kain, Paz Fernández-Valero, Silvia Castro-Cisterna, Maria Jose Arias-Tellez, Francisco B. Ortega

**Affiliations:** 1 Biomedicine Center, Laboratory of Physical Activity, Exercise and Health, Faculty of Medicine and Health Sciences, Universidad Mayor, Santiago, Chile; 2 Public Nutrition Unit, Institute of Nutrition and Food Technology (INTA), University of Chile, Santiago, Chile; 3 Carrera de Entrenador en Actividad Física y Deporte, Facultad de Ciencias Humanas, Universidad Bernardo O’Higgins, Santiago, Chile; 4 Department of Physical Education, Faculty of Education, University of the Americas, Santiago, Chile; 5 Department of Nutrition, Faculty of Medicine, University of Chile, Santiago, Chile; 6 Department of Physical Education and Sports, Faculty of Sport Sciences, Sport and Health University Research Institute (iMUDS), University of Granada, and CIBEROBN Physiopathology of Obesity and Nutrition, Granada, Spain; 7 Faculty of Sport and Health Sciences, University of Jyväskylä, Jyväskylä, Finland

**Keywords:** cardiorespiratory fitness, childhood obesity, children, fitness, girls–boys, muscular fitness, physical fitness, preschool

## Abstract

**Introduction:**

This study explores the test–retest reliability of the FITness in PREschoolers (PREFIT) battery, including cardiorespiratory fitness [20 m shuttle run test (SRT)], upper-body muscle strength (handgrip strength), lower-body explosive strength (standing long jump), speed (4 × 10 m shuttle run), and static balance (one-leg stance), in Chilean preschoolers considering weight status, sex, and age.

**Methods:**

The study included 171 Chilean preschoolers (5.4 ± 0.7 years). The PREFIT battery was applied using a test–retest design within 7–10 days between evaluations, and weight status was determined using BMI *z*-scores (ClinicalTrials.gov: NCT04269135). Statistical analyses included test–retest differences, Lin's concordance correlation coefficient, Cohen's effect size (*d*), standard error of measurement, intraclass correlation coefficient (ICC), and Bland–Altman plots. Analyses were performed using STATA.

**Results:**

Among the 171 preschoolers (5.4 ± 0.7 years), test–retest reliability ranged from excellent to moderate for handgrip strength (ICC = 0.8502; 95% CI: 0.81–0.90), 4 × 10 m shuttle run test (ICC = 0.7696, 95% CI: 0.71–0.84), and PREFIT 20 m SRT (ICC = 0.6656; 95% CI: 0.58–0.74), but was poor for the one-leg stance test (ICC = 0.46; 95% CI: 0.36–0.63). SEM and MDC95 values indicated that changes of 0.85 kg, MDC = 9,502.35 kg (handgrip strength), ≥0.89 s (4 × 10 m shuttle run test), ≥5.33 laps (20 m shuttle run test), and ≥12.43 cm (standing long jump) exceeded measurement error. Reliability was similar across sex, age, and weight-status groups. Bland–Altman analyses showed proportional bias and heteroscedasticity for the 20 m shuttle run test and one-leg stance. At the same time, the remaining tests demonstrated consistent measurement error throughout the performance spectrum.

**Conclusion:**

The PREFIT battery is appropriate for field application among Chilean preschool children, except for the one-leg stance test, which demonstrated inadequate reliability and proportional bias. Reporting MDC95 enables the interpretation of significant change within educational and intervention contexts.

## 1 Introduction

Physical fitness represents the optimal physiological function that mitigates the risk of lifestyle-related diseases linked to sedentary behavior and immobility and is essential to sustain overall health (1). It is influenced by regular physical activity, genetic makeup, and weight status (2). For this reason, physical fitness is recognized as a powerful marker of health in children and adolescents, including different physical abilities, and evaluating it in children is essential due to its relationship with health. It is strongly associated with body composition (3), adiposity, cardiometabolic disease risk, bone health, and mental and cognitive health (4). Remarkably, cardiorespiratory fitness and muscular strength are considered the most important health outcomes in the school stage and throughout adulthood, especially later in life (5). Following this, childhood aerobic fitness is strongly associated with the risk of mortality in adulthood, while muscular strength in adolescence is associated with all-cause mortality, especially cardiovascular disease and suicide (6).

Special attention has been paid to children with high socioeconomic levels, who are more likely to achieve a healthy fitness zone. Evidence indicates that children attending schools with low socioeconomic status face more barriers to increased physical fitness, associated with curriculum, teaching, school policy, and the environment (7). According to fundamental movement skills, the evidence supports a moderate-to-large positive relationship with physical fitness, with both mutually enhancing each other in preschool-aged children (8).

Assessing physical fitness in early life is important because it provides information regarding growth and development. In particular, the focus is on health and recently increased interest in studying at a preschool age. To evaluate physical fitness, it is necessary to rely on an evaluation battery corresponding to a set of physical tests that individually measure each physical capacity (i.e., cardiorespiratory fitness, muscle strength, speed, and static balance). Each battery that evaluates physical fitness must demonstrate its validity, reliability, and applicability in the population of interest (2, 9). In children and adolescents, there are a large number of batteries that measure physical fitness, which have been applied in different parts of the world, e.g., in Europe, EUROFIT (10) and ALPHA (11); in the USA, Fitnessgram (12) and PCSFN (13); in Canada, CAPL (14); in Singapore, NAPFA (15); in Japan, Physical Fitness and Athletic Ability Test (16); and in Australia, AFEA (17) batteries. Recently, experts from 50 different countries have reached an international consensus on which tests and protocols should be used in children aged 6–18 years, namely, the Youth Fitness International Test (YFIT) battery (18). However, this consensus did not include preschool-age children, because more geographically and culturally diverse evidence on reliability, validity, and health relationships needs still to be accumulated. In children, 5 years old and younger, the FITness in PREschoolers (PREFIT) battery (9) was proposed in 2016, which needs to be tested in different world regions and cultures.

The PREFIT battery consists of a series of tests that measure physical fitness and examine its relationships with health outcomes. In this context, cardiorespiratory fitness represents the ability of the cardiovascular and respiratory systems to couple when the muscles demand oxygen to maintain the intensity of physical work (19). Musculoskeletal fitness includes muscular strength, defined as the maximal capacity to generate force; muscular endurance, the ability to withstand voluntary contractions over an extended period; and explosive muscular strength or power, the ability to generate maximal strength in less time (20). Motor fitness is associated with enhanced performance in sports and other motor skills. Speed is recognized as a rapid whole-body movement (21). Agility includes speed and the ability to change directions with efficacy (22). Finally, this battery encompasses the assessment of balance, characterized as the capacity to sustain body equilibrium in both static and dynamic postures around the center of gravity (23).

The PREFIT has demonstrated reliability in the Spanish preschool population in four of the five tests, with the one-leg stance showing worse reliability (9). This finding should be confirmed or contrasted in future studies involving populations outside Europe and considering variations in weight status, such as overweight and obesity. Childhood obesity has a high prevalence worldwide (7, 8), and Chile is among the countries with the highest incidence (24), which continues to rise. According to the national nutritional report on preschoolers, 22.7% are classified as obese and 24.3% as overweight (25). This prevalence shows the negative effect of obesity on physical fitness from the first years of primary education and the exposure risk on future health (5). At present, it is unknown whether the reliability of fitness testing differs by weight status. Recent studies have analyzed this relationship in preschoolers with excess weight (10), highlighting the need for reliable and reproducible fitness measurement tools for these preschool children. Replication of reliability studies in populations with different characteristics and in other parts of the world is necessary, especially for intervention studies assessing pre- and post-intervention changes or for prospective cohort studies. It has been reported that children in different stages of educational level from Chile have low levels of physical activity (26) and a high prevalence of overweight or obesity (50.6%) (25), which might impact their fitness levels and motor skills to perform fitness testing. It is, therefore, of interest to test the reliability of fitness tests in the Chilean pediatric population. Furthermore, similar and reliable battery physical fitness tests will analyze the physical fitness results in different countries. This study aims to determine the test–retest reliability of the PREFIT fitness-test battery in Chilean preschoolers and to examine whether it differs by weight status, sex, or age.

## 2 Method

### 2.1 Participants and study design

An observational reliability study with a cross-sectional design was conducted, applying the physical tests included in the PREFIT battery (9) and assessing the test–retest method, with measurements taken within 7–10 days. Six schools participated in this study. The evaluators received training through both theoretical and practical sessions on test administration. Each group of three evaluators was supervised by evaluation coordinators (PF-V, SC-C, MA-T), who had extensive prior experience in evaluating physical condition and anthropometric variables. The coordinators, in turn, were supervised by the project director (JS-S), who ensured compliance with previously published protocols (FO). Each team evaluated two educational institutions, both in the test and in the retest.

This study was conducted as part of the research network “Preschool Fit-healthy and Smart: PREFIT-Chile,” ClinicalTrials.gov Identifier: NCT04269135.

The study included 171 preschoolers from six schools in the Valparaíso Region, Chile, participating in the Ministry of Sports “Play and Learn” Program, which provides physical education twice a week delivered by physical education teachers. This program is part of the Ministry of Sports initiative, “Growing in Motion,” and has been implemented since 2014 in vulnerable schools. In this context, these schools are defined as those institutions that serve a significant number of children in situations of socioeconomic and social vulnerability, as measured by national indices such as the “National Household Registry” determined by the Ministry of Social Development (27) or the “School Multidimensional Vulnerability Index” (28) defined by the Ministry of Education. This study was approved by the Human Ethics and Research Committee of the Institute of Nutrition and Food Technology Dr. Fernando Monckeberg Barros (INTA) of the University of Chile (registration 9-2019).

### 2.2 Instruments

Anthropometric variables: Weight status was determined using BMI z-scores according to the WHO reference values. Weight (kg) was measured with a SECA scale model 813 and height (cm) with a portable altimeter SECA model 213. BMI was calculated using the weight/height^2^ equation (29).

**PREFIT battery** consists of five fitness tests previously shown to be reliable in preschoolers (9): (i) Cardiorespiratory fitness was measured with the PREFIT 20 m shuttle run test (PREFIT 20 m SRT), a test adapted for preschoolers following a sound stimulus. The number of laps performed was recorded. (ii) Upper-body muscular strength was measured by the maximum handgrip strength with an analogous dynamometer TKK5001. For the test, children stand with both feet on the ground and their tested arm straight down. Their shoulders are slightly apart (approximately 10 °, not touching their body), elbows are fully extended, forearms are neutral, and wrists are without flexion (30). Both the grip of the right and left hands is measured, with two repetitions in each, being considered the highest value of each hand. The mean value was recorded in kg. (iii) The standing long jump test was used to measure lower-body muscular strength: On a flat surface, three attempts were made, and the maximum value of the distance reached was recorded with a tape measure; the unit used for this measurement was cm. (iv) The 4 × 10 m shuttle run test (4 × 10 m SRT) measured speed-agility. A flat surface, a stopwatch, and marker cones were required. This test was performed twice, and the shortest time was recorded. (v) Static balance was measured by the one-leg stance test, requiring a flat surface and a stopwatch. The balance time was timed with the left leg and then with the right leg, and the average of both feet was recorded (31).

### 2.3 Administration PREFIT battery

This work is part of The PREFIT Project (https://profith.ugr.es/en/projects/prefit/) and employs a comparable methodology for test administration and equipment calibration. However, we created a PREFIT-Chile operation manual (Supplementary material) and a video detailing how to replicate this test: https://youtu.be/XiqsEjB2Hnc. Before applying this battery, the children engaged in a warm-up activity involving a motor game, specifically “to play tag,” for 5 min. Furthermore, they participated in a motor story that facilitated the administration of physical assessments. In this context, a Chilean adaptation of the motor story has been developed in Spanish, incorporating specific movements to evaluate each test, “Sam and Samantha in the Amazon Jungle.” The narrative emphasizes the physical tests through playful interactions.

### 2.4 Statistical analysis

For statistical analysis, the variables were presented as mean and standard deviation. Normality was assessed using the Shapiro–Wilk test and histogram. Differences in values between the retest and test were analyzed using the Student’s *t*-test of dependent means. In addition, two-way analysis of variance (ANOVA) was used to investigate the influence of sex, age, and weight status.

To determine the agreement between the retest and test, Lin's concordance correlation coefficient (32), Cohen's *d* effect size (32), and typical error were calculated (33). The reliability of physical tests was evaluated using mixed-effects models, incorporating the educational institution as a random intercept to account for the hierarchical structure of the data. The intraclass correlation coefficient (ICC) (2,1) was computed based on a two-way random-effects model designed to assess absolute agreement for single measurements. The estimates were accompanied by 95% confidence intervals to quantify the reliability across measurements. Based on the ICC, the standard error of measurement [SEM = SDpooled·√(1–ICC)] and the minimum detectable change at 95% (MDC95 = SEM × 1.96 × √2) were computed as indicators of measurement precision and the minimum change required to signify a genuine variation in performance. Similarly, the coefficient of variation percentage (CV%) was calculated for the tests based on time or distance to characterize the relative variability of the measurements. For the agreement analysis, the Bland–Altman method (33) was applied to assess the presence of proportional bias by regressing the test–retest difference on the mean of each measurement pair. All analyses were performed on both the total sample and stratified by sex, age, and nutritional status, to explore possible variations in reliability patterns between subgroups.

Cohen’s *d* effect size indicates that values <0.20 are considered powerful, whereas values of 0.5 are considered moderate. The typical error evaluates the variation from one measurement to another. Intraclass correlation (ICC) was used to interpret the method's reliability qualitatively: values of 0.75–0.90 are considered excellent, 0.5–0.75 moderate, and <0.5 poor (34). Analyses were performed with STATA version 16.0 (StataCorp LLC, College Station, TX, USA) and GraphPad Prism version 9 (GraphPad Software, CA, USA).

## 3 Results

**Figure 1:**
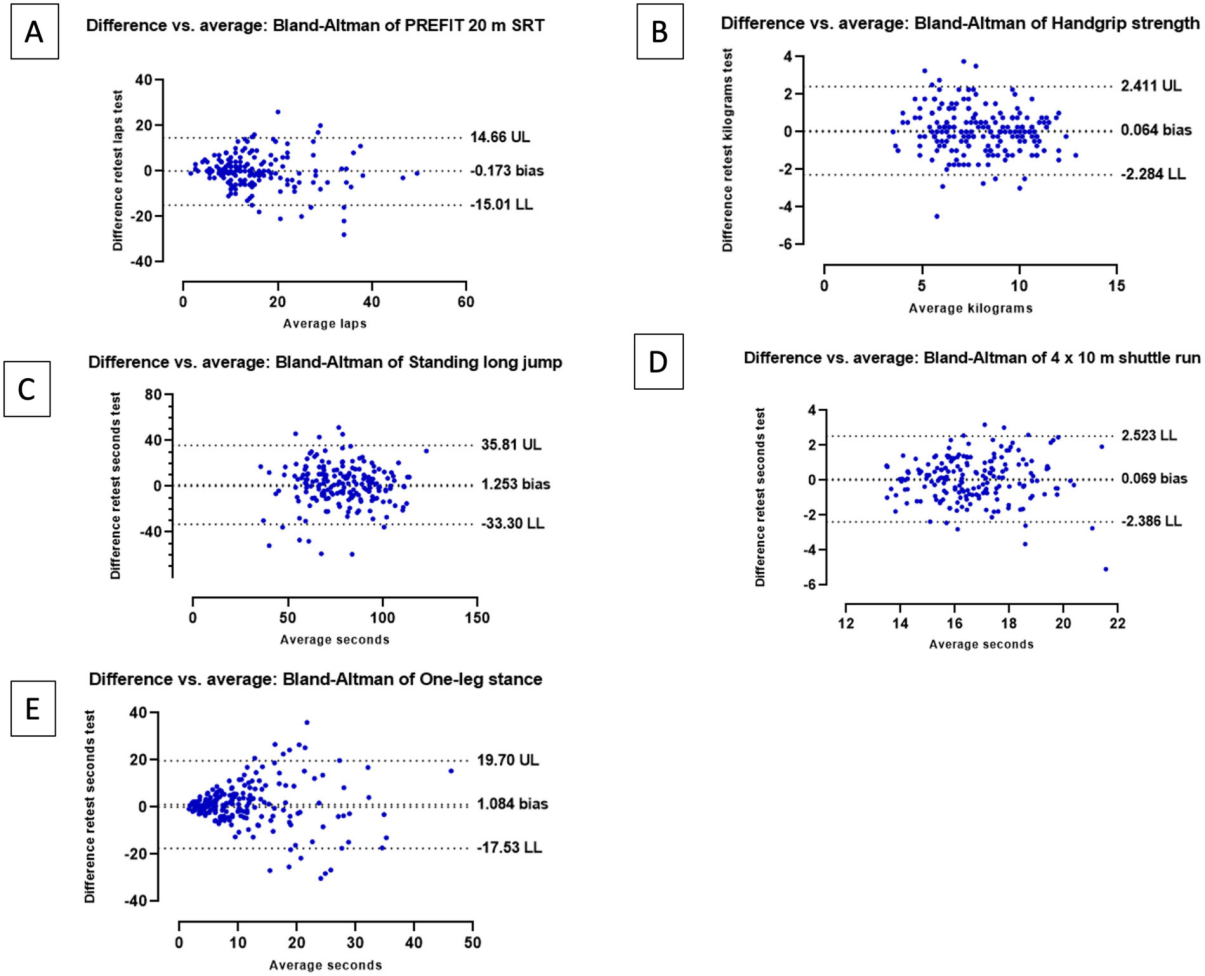
Bland–Altman plots of the PREFIT battery validation. **(A)** The agreement between pre- and post-20 m SRT, **(B)** handgrip strength, **(C)** standing long jump, **(D)** 4 × 10 m SRT, and **(E)** one-leg stance. A central dotted line represents the mean differences (retest and test). The upper and lower 95% limits of agreement (mean differences ± 1.96 SD of the differences) are represented by the upper and lower dotted lines.

Table 1 describes the characteristics of the 171 preschoolers who participated (60.2% boys and 39.8% girls) with an age range between 4 and 6 years (5.4 ± 0.7 years). BMI/age showed that 48.4% of preschoolers had a normal weight, while 51.6% had excessive weight (28.6% overweight and 23% obese). Figure 1 provides additional details regarding participant flow.

**Table 1:** Anthropometric characteristics of the studied population.

Anthropometry	All (*n* = 171)	Boys (*n* = 103)	Girls (*n* = 68)
	Mean ± SD	Mean ± SD	Mean ± SD
Age (years)^a^	5.4 ± 0.7	5.4 ± 0.6	5.5 ± 0.7
Weight (kg)^a^	21.7 ± 4.0	21.6 ± 3.7	21.2 ± 4.1
Height (cm)^a^	111.6 ± 5.8	111.5 ± 5.7	110.8 ± 5.8
BMI (kg/m2)^a^	17.3 ± 2.1	17.3 ± 2.0	17.2 ± 2.2
Weight status^b^^,^^c^			
Normal	48.4%	44.2%	52.3%
Overweight	28.6%	27.9%	33.9%
Obesity	23.0%	27.9%	13.9%

BMI, body mass index.

a Data are expressed as a mean ± SD.

b Data are expressed in *n* and %.

c Classification with the *z*-scores of the body mass index according to the WHO growth reference for school-aged children and adolescents (29).

Table 2 presents the reliability and concordance statistics for the retest and test administrations of the physical fitness tests included in the PREFIT battery for preschool children. Results indicate that the PREFIT battery physical fitness tests are reliable for preschoolers (20 m shuttle run, ICC = 0.6656; handgrip strength, ICC = 0.8502; standing long jump, ICC = 0.6736; and 4 × 10 m shuttle run, ICC = 0.7696). The one-leg stance showed the worst reliability among the tests studied (ICC = 0.4602).

**Table 2:** Test and retest and feasibility and reliability of PREFIT physical fitness tests.

Physical fitness test	Test (*n* = 171)	Retest (*n* = 171)	Diff (retest minus test) (*n* = 171)	*p* ^a^	LC	Effect size	Typical error	ICC
	Mean ± SD	Mean ± SD	Mean ± SD					
PREFIT 20 m SRT (laps)^b^	15.0 ± 8.9	15.2 ± 9.8	0.2 ± 7.6	0.7599	0.673	0.032	5.35	0.6656
Handgrip strength (kg)^b^	8.0 ± 2.1	7.9 ± 2.2	−0.1 ± 1.2	0.4519	0.844	0.075	0.85	0.8502
Standing long jump (cm)^b^	79.3 ± 19.2	77.8 ± 19.4	−1.3 ± 17.6	0.2562	0.682	0.115	12.47	0.6736
4 × 10 m shuttle run (s)^b^	16.7 ± 1.9	16.6 ± 1.8	−0.1 ± 1.3	0.4485	0.749	0.566	0.89	0.7696
One-leg stance (s)^b^	11.9 ± 9.1	10.8 ± 9.4	−1.1 ± 9.5	0.1080	0.466	0.161	6.72	0.4602

LC, Lin's concordance correlation coefficient; effect size, Cohen's *d* effect size; ICC, intraclass correlation.

a One-sample *t*-test (mean different from 0 for all measures).

b Data are expressed as a mean ± SD.

The Bland–Altman graphs presented in Figure 1 show the random and systematic variability of these results as the magnitude of the measurement increases. Figure 1A shows a certain degree of heteroscedasticity in the PREFIT 20 m SRT, which is even more marked in the case of the one-leg stance test (Figure 1E), indicating more test–retest variability (i.e., poorer reliability) as children perform better in these tests. This pattern was not clearly observed in the other tests.

Table 3 shows the reliability results observed in the test–retest and analyzed by sex; no differences were observed in any PREFIT battery tests. Table 4 shows the statistics of the test–retest statistics by age group. In this context, no differences were found in the results of the physical fitness tests. Similarly, no differences were observed by age. Table 5 shows the reliability results according to weight status. We observed that the *p*-value of the test–retest difference is not significant in physical fitness tests, nor according to weight status.

**Table 3:** Test–retest of PREFIT physical fitness tests for boys and girls.

Physical fitness test	Boys (*n* = 103)		Girls (*n* = 68)		Mean differences (retest minus test)				Sex-effect *p*-ANOVA^a^
	Test	Retest	Test	Retest	Boys	*p* ^b^	Girls	*p* ^b^	
	Mean ± SD	Mean ± SD	Mean ± SD	Mean ± SD					
PREFIT 20 m shuttle run (laps)^c^	16.2 ± 9.3	16.5 ± 10.7	13.4 ± 8.1	13.3 ± 8.0	0.4 ± 8.3	0.6599	−0.1 ± 6.3	0.8967	0.6860
Handgrip strength (kg)^c^	8.0 ± 2.2	8.0 ± 2.3	8.0 ± 1.9	7.9 ± 2.2	−0.1 ± 1.3	0.5241	−0.1 ± 1.0	0.5774	0.8514
Standing long jump (cm)^c^	80.7 ± 19.2	79.3 ± 19.7	77.2 ± 19.1	75.6 ± 18.8	−1.0 ± 17.2	0.4133	−1.6 ± 18.3	0.4334	0.7100
4 × 10 m shuttle run(s)^c^	16.7 ± 1.9	16.6 ± 1.9	16.7 ± 1.8	16.6 ± 1.5	−0.1 ± 1.3	0.7359	−0.1 ± 1.2	0.4108	0.6890
One-leg stance (s)^a^	11.8 ± 9.3	10.1 ± 9.2	11.9 ± 8.8	11.8 ± 9.5	−1.7 ± 9.4	0.0510	−0.2 ± 9.6	0.7116	0.2994

Mean differences were entered as dependent variables and sex (i.e., boys and girls) and age groups as fixed factors.

a Two-way ANOVA.

b One-sample *t*-test (mean different from 0 for all measures).

c Data are expressed as a mean ± SD.

**Table 4:** Test–retest and mean differences of PREFIT physical fitness tests analyzed by age.

Physical fitness test	4 years (*n* = 50)		5 years (*n* = 84)		6 years (*n* = 37)		Mean differences (retest minus test)						Age-effect *p*-ANOVA^a^
	Test	Retest	Test	Retest	Test	Retest	4 years	*p* ^b^	5 years	*p* ^b^	6 years	*p* ^b^	
	Mean ± SD	Mean ± SD	Mean ± SD	Mean ± SD	Mean ± SD	Mean ± SD							
PREFIT 20 m shuttle run (laps)	11.2 ± 5.7	10.9 ± 5.9	15.4 ± 8.2	16.2 ± 10.5	19.2 ± 11.7	18.5 ± 10.4	−0.3 ± 5.7	0.7513	0.8 ± 8.0	0.3634	−0.6 ± 8.6	0.6471	0.5550
Handgrip strength (kg)	6.6 ± 1.6	6.3 ± 1.8	8.1 ± 1.9	8.0 ± 2.0	9.4 ± 1.9	9.6 ± 1.8	−0.2 ± 1.1	0.1299	−0.1 ± 1.2	0.4955	0.7 ± 1.3	0.3782	0.2594
Standing long jump (cm)	74.6 ± 19.7	71.0 ± 20.1	79.1 ± 19.2	78.2 ± 17.3	84.6 ± 17.7	85.4 ± 20.3	−3.6 ± 15.4	0.0948	−0.9 ± 19.7	0.5242	0.7 ± 15.0	0.7448	0.4429
4 × 10 m shuttle run(s)	17.7 ± 1.9	17.6 ± 1.5	16.5 ± 1.7	16.5 ± 1.8	15.8 ± 1.4	15.7 ± 1.5	−0.1 ± 1.1	0.4255	−0.0 ± 1.4	0.9927	−0.1 ± 1.0	0.4287	0.8224
One-leg stance (s)	6.9 ± 5.0	5.4 ± 3.9	12.4 ± 8.8	11.2 ± 8.5	16.3 ± 10.6	16.0 ± 12.1	−1.5 ± 5.6	0.0516	−1.2 ± 10.2	0.2391	−0.3 ± 11.5	0.8446	0.8615

The values are expressed as a mean ± SD.

a Two-way ANOVA. Mean differences were entered as dependent variables and sex and groups of age (i.e., 4, 5, and 6) as fixed factors.

b One-sample *t*-test (mean different from 0 for all measures).

**Table 5:** Test–retest and mean differences of PREFIT physical fitness tests divided by nutritional status.

Physical fitness test	Normal weight (*n* = 83)		Overweight (*n* = 49)		Obese (*n* = 39)		Mean differences (retest minus test)						WS-effect *p*-ANOVA^a^
	Test	Retest	Test	Retest	Test	Retest	Normal weight	*p* ^b^	Overweight	*p* ^b^	Obese	*p* ^b^	
	Mean ± SD	Mean ± SD	Mean ± SD	Mean ± SD	Mean ± SD	Mean ± SD							
PREFIT 20 m shuttle run (laps)	19.6 ± 11.2	18.8 ± 12.8	15.1 ± 7.1	16.1 ± 10.0	14.6 ± 7.8	14.1 ± 6.7	−0.8 ± 9.7	0.4018	1.0 ± 6.5	0.4018	−0.5 ± 6.7	0.7354	0.6326
Handgrip strength (kg)	8.4 ± 1.8	8.4 ± 2.0	8.4 ± 2.0	8.7 ± 1.9	9.5 ± 1.8	9.6 ± 2.0	−0.1 ± 1.2	0.5689	0.2 ± 1.5	0.3719	0.1 ± 1.1	0.5131	0.4773
Standing long jump (cm)	84.7 ± 20.3	83.9 ± 18.6	76.0 ± 17.3	76.2 ± 15.0	81.3 ± 16.4	76.5 ± 22.6	−0.8 ± 21.4	0.7735	0.1 ± 16.2	0.9648	−4.8 ± 13.4	0.0719	0.5243
4 × 10 m shuttle run(s)	15.9 ± 1.7	15.8 ± 1.6	16.7 ± 1.6	16.9 ± 2.1	16.5 ± 1.8	16.4 ± 1.4	−0.2 ± 1.2	0.3166	0.2 ± 1.6	0.5068	−0.1 ± 1.3	0.8127	0.5074
One-leg stance (s)	14.3 ± 9.0	15.3 ± 11.1	13.6 ± 8.7	10.7 ± 8.3	12.8 ± 7.8	10.9 ± 8.3	1.0 ± 11.4	0.4969	−2.8 ± 8.5	0.0540	−1.9 ± 7.5	0.1926	0.1563

The values are expressed as a mean ± SD.

WS, weight status.

a Two-way ANOVA. Mean differences were entered as dependent variables and sex and nutritional status (i.e., normal weight, overweight, and obese) as fixed factors.

b One-sample *t*-test (mean different from 0 for all measures).

## 4 Discussion

The findings of the present study suggest that the PREFIT battery (9) had moderate to excellent reliability in Chilean preschoolers aged 4, 5, and 6 years for the PREFIT 20 m SRT, handgrip strength, standing long jump, and 4 × 10 m SRT, yet poor reliability was observed for the one-leg stance test. When comparing the results with a previous study (9) testing the reliability of the PREFIT battery in Spanish preschoolers, we observed a lower Lin's concordance correlation coefficient for most of the tests in the Chilean compared with Spanish children. Moreover, the standard deviation of the test–retest difference was larger in Chilean children than that in the Spanish children for the PREFIT 20 m SRT and the standing long jump test, indicative of a poorer agreement between measurements, whereas it was similar for the handgrip strength and 4 × 10 m SRT and smaller for the one-leg stance test. Both studies showed consistently that the one-leg stance test had the poorest reliability among the tests included in the PREFIT fitness-test battery in preschoolers, and its usefulness in this age group is therefore highly questionable once the results have been confirmed in children from different parts of the world and with different characteristics (e.g., % of overweight/obesity). Consistent with previous research, the one-leg stance test showed poor reliability and significant heteroscedasticity, raising questions about its usefulness in 4–6-year-olds. We suggest (i) refining the protocol (fixed gaze target, arms akimbo, standardized footwear, limited trial duration), (ii) additional familiarization, and (iii) considering other balance tasks that are more suitable for this age group (e.g., simplified tandem stance) in school settings.

In addition to confirming/contrasting the reliability values of the previous studies, a unique contribution of this study was to investigate whether the reliability of fitness tests in preschoolers differed by weight status, which was not previously studied. Our findings support that the reliability of the test does not differ by weight status, nor by sex or age groups, which is good news and suggests the idea of a fitness-test battery to be used in school settings for all children equally.

Regarding the reliability results of the PREFIT 20 m SRT, our results support moderate reliability according to the ICC values. This test is easy to apply in schools that have enough space. However, some kindergartens do not have a 20 m linear space to apply this test, which reduces its feasibility in school settings. However, this is cultural- and country-specific. The handgrip strength test presents excellent reliability, similar to the study by Cadenas-Sanchez et al. (9). The standing long jump test showed moderate reliability, which was also in line with the results obtained in the Spanish population (9).

In our study, the 4 × 10 m SRT test demonstrated excellent reliability, also in line with Cadenas-Sanchez et al. (9). There are few instruments and tools to assess physical fitness in this age group. Recently, the need for these measures to be reproducible, comparable, and reliable to identify the different characteristics between populations adequately has been highlighted (35, 36), and to be easy and low-cost to administrate on the physical space at school. Unlike laboratory tests (37), which allow measurement with great accuracy, they only allow a small number of participants to be reached and need equipment, which is sometimes expensive. In that sense, our results, together with those from Cadenas-Sanchez et al. (9), support that the PREFIT battery is overall reliable to be used in preschoolers, except for the one-leg stance test. Other studies of reliability in preschoolers using some tests of the PREFIT battery is the “Fuprecol kids” battery demonstrated substantial reliability by ICC, and where of five, only four tests were used (20 m SRT, handgrip strength, standing long jump, and 4 × 10 m SRT: ICC = 0.944, 0.941, 0.957, and 0.978, respectively) on 86 preschoolers (48 boys and 42 girls). However, this study did not analyze the results according to weight status (35). The study presented by Fang and Ho (38) which analyzed the reliability and reproducibility of the Chinese battery “National Physical Fitness” demonstrated high reproducibility by ICC on 209 preschoolers (111 boys and 98 girls, from 3.5 to 6 years old), where the only test that is similar to the PREFIT battery is the standing long jump (ICC = 0.96). However, showing differences between retest and test, these analyses were only performed considering age and sex, but did not include anthropometric variables. Therefore, having a reliable battery for children according to weight status is relevant since it has been reported that over 340 million children and adolescents, and 39 million children of preschool age, were overweight pre-pandemic (24) and post-pandemic in 2022, around the world. The number of boys and girls with obesity reached 65.1 and 94.2 million, respectively (39). Countries such as China, Indonesia, and Brazil expect 2030 figures to exceed one million children with obesity (40). From this perspective, it was necessary to investigate whether the reliability of fitness testing differed by weight status. The results from our study suggest that this is not the case, at least in preschoolers, yet future studies should confirm/contrast our findings in this same age group, as well as in older children and adolescents.

Although fitness levels differ by sex, our study showed that the PREFIT battery is equally reliable and reproducible for both boys and girls. Similarly, no differences were found between the ages of 4, 5, and 6 years in reliability in our study. These findings support consistency by sex and age, aligning with the study by Cadenas-Sanchez et al. (9) on Spanish preschoolers.

The strength of this study lies in its necessity; the claim that the PREFIT test battery should be validated across different samples is well-founded.

The limitations of our study include that the results may not be generalizable to settings beyond public schools within a national program targeting vulnerable communities. Inter-rater reliability was not directly evaluated. The 7–10-day retest window could potentially introduce learning or maturation effects. Furthermore, environmental conditions were neither fully standardized nor modeled. Attention should be given to clustering by school and proportional bias in future surveillance efforts. Furthermore, the analysis was conducted with children aged 4, 5, and 6 years, excluding children who were 3 years old, a group also regarded as preschool age. Another aspect to consider in this study is its straightforward design, which provides for a single sample, a single test battery, and a focus on a single key metric—reliability. This simplicity opens up valuable discussions about the adequacy of these elements in providing comprehensive insights. Exploring additional dimensions could deepen and broaden the applicability of the findings.

## 5 Conclusion

In summary, our findings indicate that the PREFIT fitness-test battery is a dependable assessment tool for evaluating physical fitness in Chilean preschool children, with the notable exception of the one-leg stance test, which demonstrated inadequate reliability and proportional bias. Additionally, the test battery demonstrated strong reliability across various demographic groups, including different sexes, ages, and weight statuses. Together, reporting MDC95 enables the interpretation of significant change within educational and intervention contexts. This consistency in results significantly enhances its potential for use in school environments, where understanding children's fitness levels is essential for promoting healthy development and activity.

## Data Availability

The raw data supporting the conclusions of this article will be made available by the authors, without undue reservation.

## References

[1] Donnelly JE Hillman CH Castelli D Etnier JL Lee S Tomporowski P Physical activity, fitness, cognitive function, and academic achievement in children: a systematic review. Med Sci Sports Exerc. (2016) 48(6):1197–222. 10.1249/MSS.0000000000000901 27182986 PMC4874515

[2] Soto-Sanchez J. Pruebas y medidas de la condición física. In: Ferrari G Mehecha-Matsudo S, editors. Actividad Física y Ejercicio en la Salud del Niño y el Adolescente. Santiago, Chile: Mediterraneo (2021). p. 300.

[3] Henriksson P Sandborg J Henstrom M Delisle Nystrom C Ek E Ortega FB Body composition, physical fitness and cardiovascular risk factors in 9-year-old children. Sci Rep. (2022) 12(1):2665. 10.1038/s41598-022-06578-w 35177687 PMC8854391

[4] Tomkinson GR Carver KD Atkinson F Daniell ND Lewis LK Fitzgerald JS European Normative values for physical fitness in children and adolescents aged 9–17 years: results from 2 779 165 eurofit performances representing 30 countries. Br J Sports Med. (2018) 52(22):1445–56. 10.1136/bjsports-2017-098253 29191931

[5] Högström G Nordström A Nordström P. Aerobic fitness in late adolescence and the risk of early death: a prospective cohort study of 1.3 million Swedish men. Int J Epidemiol. (2016) 45(4):1159–68. 10.1093/ije/dyv321 26686843

[6] Ortega FB Silventoinen K Tynelius P Rasmussen F. Muscular strength in male adolescents and premature death: cohort study of one million participants. Br Med J. (2012) 345:e7279. 10.1136/bmj.e7279 23169869 PMC3502746

[7] Peralta LR Mihrshahi S Bellew B Reece LJ Hardy LL. Influence of school-level socioeconomic status on children’s physical activity, fitness, and fundamental movement skill levels. J Sch Health. (2019) 89(6):460–7. 10.1111/josh.12761 30945311

[8] Chen J Song W Zhao X Lou H Luo D. The relationship between fundamental motor skills and physical fitness in preschoolers: a short-term longitudinal study. Front Psychol. (2023) 14:1270888. 10.3389/fpsyg.2023.1270888 37780141 PMC10536265

[9] Cadenas-Sanchez C Martinez-Tellez B Sanchez-Delgado G Mora-Gonzalez J Castro-Pinero J Lof M Assessing physical fitness in preschool children: feasibility, reliability and practical recommendations for the PREFIT battery. J Sci Med Sport. (2016) 19(11):910–5. 10.1016/j.jsams.2016.02.003 26947061

[10] Council of Europe Committee of Experts on Sports R. EUROFIT: Handbook for the EUROFIT Tests of Physical Fitness. 2nd ed. Strasbourg: Sports Division Strasbourg, Council of Europe Publishing and Documentation Service (1993).

[11] Espana-Romero V Artero EG Jimenez-Pavon D Cuenca-Garcia M Ortega FB Castro-Pinero J Assessing health-related fitness tests in the school setting: reliability, feasibility and safety; the ALPHA study. Int J Sports Med. (2010) 31(7):490–7. 10.1055/s-0030-1251990 20432194

[12] Meredith MD Welk G Institute C. Fitnessgram and Activitygram Test Administration Manual-Updated, 4th ed. Dallas, TX: Human Kinetics (2010).

[13] PCFSN (President’s Council on Fitness, and Nutrition). The president’s challenge: physical activity & fitness awards program 2010–2011. Available online at: https://gilmore.gvsd.us/documents/Info/Forms/Teacher%20Forms/Presidentialchallengetest.pdf (Accessed May 05, 2019).

[14] Lloyd M Colley RC Tremblay MS. Advancing the debate on ‘fitness testing’ for children: perhaps we're riding the wrong animal. Pediatr Exerc Sci. (2010) 22(2):176–82. 10.1123/pes.22.2.176 20567039

[15] Schmidt GJ. Muscular endurance and flexibility components of the Singapore National Physical Fitness Award. Aust J Sci Med Sport. (1995) 27(4):88–94. 8833185

[16] Shingo N Takeo M. The educational experiments of school health promotion for the youth in Japan: analysis of the ‘sport test’ over the past 34 years. Health Promot Int. (2002) 17(2):147–60. 10.1093/heapro/17.2.147 11986296

[17] Australian Council for Health PE, Recreation, Healthy Lifestyles Bookshop A, Education FM. Australian Fitness Education Award: Teacher’s Handbook & Curriculum Ideas. Hindmarsh: ACHPER (1996).

[18] Ortega FB Zhang K Cadenas-Sanchez C Tremblay MS Jurak G Tomkinson GR The Youth Fitness International Test (YFIT) battery for monitoring and surveillance among children and adolescents: a modified Delphi consensus project with 169 experts from 50 countries and territories. J Sport Health Sci. (2025) 14:101012. 10.1016/j.jshs.2024.101012 39577493 PMC11863322

[19] Dafoe W. Principles of exercise testing and interpretation. Can J Cardiol. (2007) 23(4):274. 10.1016/S0012-3692(16)31282-X

[20] Ortega FB Cadenas-Sánchez C Sánchez-Delgado G Mora-González J Martínez-Téllez B Artero EG Systematic review and proposal of a field-based physical fitness-test battery in preschool children: the PREFIT battery. Sports Med. (2015) 45(4):533–55. 10.1007/s40279-014-0281-8 25370201

[21] Sheppard JM Young WB. Agility literature review: classifications, training and testing. J Sports Sci. (2006) 24(9):919–32. 10.1080/02640410500457109 16882626

[22] Caspersen CJ Powell KE Christenson GM. Physical activity, exercise, and physical fitness: definitions and distinctions for health-related research. Public Health Rep. (1985) 100(2):126–31. 3920711 PMC1424733

[23] Melo RS Marinho S Freire MEA Souza RA Damasceno HAM Raposo MCF. Static and dynamic balance of children and adolescents with sensorineural hearing loss. Einstein (Sao Paulo). (2017) 15(3):262–8. 10.1590/s1679-45082017ao3976 29091145 PMC5823037

[24] Collaboration NCDRF. Worldwide trends in body-mass index, underweight, overweight, and obesity from 1975 to 2016: a pooled analysis of 2416 population-based measurement studies in 128.9 million children, adolescents, and adults. Lancet. (2017) 390(10113):2627–42. 10.1016/S0140-6736(17)32129-3 29029897 PMC5735219

[25] Becas JNdAEy. In: Datos SdEyAd, editor. Informe Ejecutivo: Resultados Mapa Nutricional 2023. Santiago: Junta Nacional de Auxilio Escolar y Becas (2024). p. 28.

[26] Tremblay MS Barnes JD Demchenko I Gonzalez SA Brazo-Sayavera J Kalinowski J Active healthy kids global alliance global matrix 4.0—a resource for physical activity researchers. J Phys Act Health. (2022) 19(11):693–9. 10.1123/jpah.2022-0257 36280231

[27] Gobierno de Chile. Registro Social de Hogares. Available online at: https://www.ventanillaunicasocial.gob.cl/registro-social-hogares (Accessed December 01, 2025).

[28] Junta Nacional Escolar de Auxilios y Becas. Ministerio de Educación. Índices de Vulnerabilidad. Available online at: https://www.junaeb.cl/medicion-la-vulnerabilidad-ivm/#:∼:text=Medición%20de%20la%20Vulnerabilidad%20Multidimensional%20del%20Estudiante%20%7C%20Junaeb (Accessed December 01, 2025).

[29] de Onis M Onyango AW Borghi E Siyam A Nishida C Siekmann J. Development of a WHO growth reference for school-aged children and adolescents. Bull World Health Organ. (2007) 85(9):660–7. 10.2471/BLT.07.043497 18026621 PMC2636412

[30] Sanchez-Delgado G Cadenas-Sanchez C Mora-Gonzalez J Martinez-Tellez B Chillon P Lof M Assessment of handgrip strength in preschool children aged 3 to 5 years. J Hand Surg Eur. (2015) 40(9):966–72. 10.1177/1753193415592328 26141024

[31] Cadenas-Sanchez C Alcantara-Moral F Sanchez-Delgado G Mora-Gonzalez J Martinez-Tellez B Herrador-Colmenero M Assessment of cardiorespiratory fitness in preschool children: adaptation of the 20 metres shuttle run test. Nutr Hosp. (2014) 30(6):1333–43. 10.3305/nh.2014.30.6.7859 25433116

[32] Portney LG. Foundations of Clinical Research. Applications to Evidence-Based Practice. 4 ed. United States of America: F.A. DAVIS (2020).

[33] Hopkins WG. Measures of reliability in sports medicine and science. Sports Med. (2000) 30(1):1–15. 10.2165/00007256-200030010-00001 10907753

[34] Liljequist D Elfving B Skavberg Roaldsen K. Intraclass correlation—a discussion and demonstration of basic features. PLoS One. (2019) 14(7):e0219854. 10.1371/journal.pone.0219854 31329615 PMC6645485

[35] Amado-Pacheco JC Prieto-Benavides DH Correa-Bautista JE Garcia-Hermoso A Agostinis-Sobrinho C Alonso-Martinez AM Feasibility and reliability of physical fitness tests among Colombian preschool children. Int J Environ Res Public Health. (2019) 16(17):3069. 10.3390/ijerph16173069 31450815 PMC6747194

[36] Cadenas-Sanchez C Leger L. Enhancing the discussion on physical fitness assessment in preschool children: a focus on the PREFIT battery. Am J Hum Biol. (2024) 36(2):e24014. 10.1002/ajhb.24014 37964447

[37] King-Dowling S Fortnum K Chirico D Le T Kwan MYW Timmons BW Reliability of field- and laboratory-based assessments of health-related fitness in preschool-aged children. Am J Hum Biol. (2024) 36(2):e23987. 10.1002/ajhb.23987 37725014

[38] Fang H Ho IMK. Intraday reliability, sensitivity, and minimum detectable change of national physical fitness measurement for preschool children in China. PLoS One. (2020) 15(11):e0242369. 10.1371/journal.pone.0242369 33216780 PMC7678998

[39] Phelps NH Singleton RK Zhou B Heap RA Mishra A Bennett JE Worldwide trends in underweight and obesity from 1990 to 2022: a pooled analysis of 3663 population-representative studies with 222 million children, adolescents, and adults. Lancet. (2024) 403(10431):1027–50. 10.1016/S0140-6736(23)02750-2 38432237 PMC7615769

[40] Jebeile H Kelly AS O'Malley G Baur LA. Obesity in children and adolescents: epidemiology, causes, assessment, and management. Lancet Diabetes Endocrinol. (2022) 10(5):351–65. 10.1016/S2213-8587(22)00047-X 35248172 PMC9831747

