# Supplementary material for: Reliability of the PREFIT fitness-test battery in Chilean preschoolers
Source: Front Pediatr. 2026 Jan 12;13:1654731. doi: 10.3389/fped.2025.1654731 (PMC12833291; doi:10.3389/fped.2025.1654731)
Supplement: Supplementary file 1 [file Supplementaryfile1.pdf]

PREFIT | Chile

Centro de BioMedicina
Temuco - Santiago
Universidad Mayor

Universidad de Granada

Universidad de Chile
Instituto de Nutrición y Tecnología de los Alimentos
Doctor Fernando Monckeberg Barros

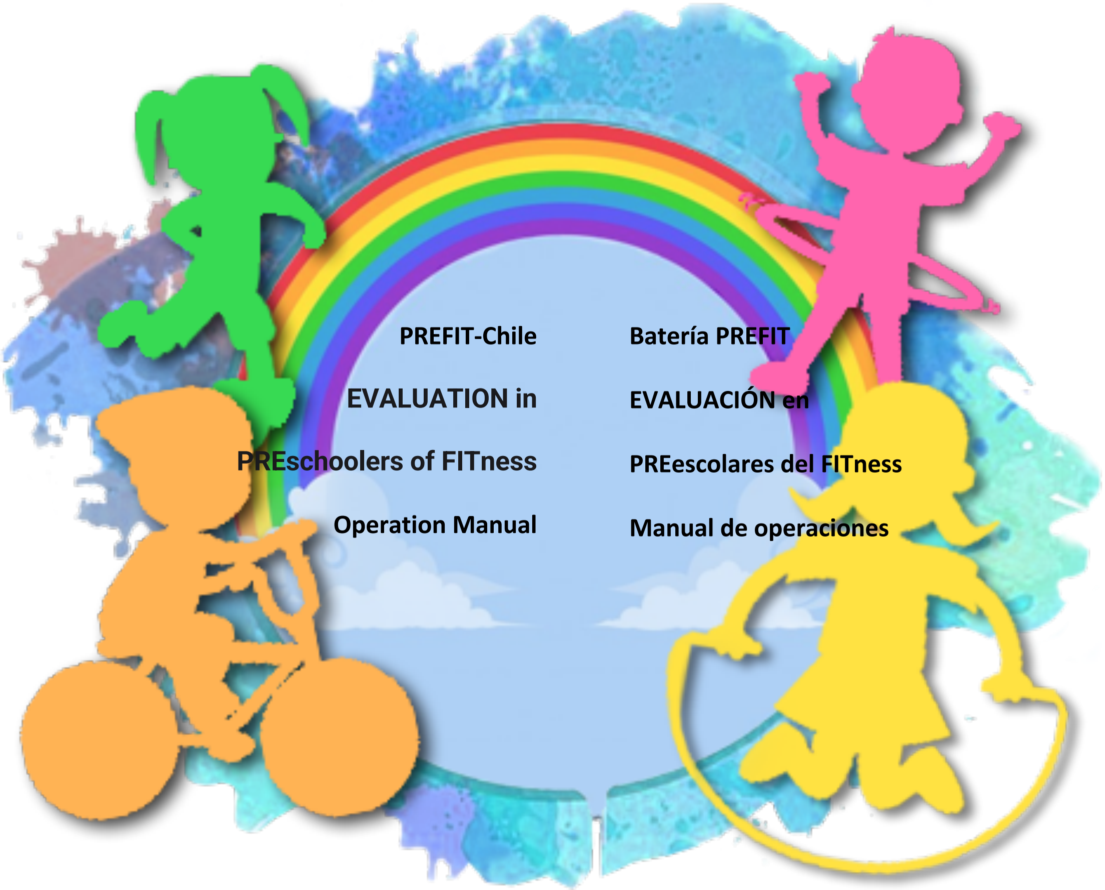

# PREFIT-Chile
# EVALUATION in PREschoolers of FITness
# Operation Manual

# Batería PREFIT
# EVALUACIÓN en PREescolares del FITness
# Manual de operaciones

# Introduction

This document aims to assist and guide the implementation of the "PREFIT Battery: Fitness Assessment in Preschoolers." It is prepared as an adaptation of the original protocol of the "PREFIT Battery: Fitness Assessment in Preschoolers" from the University of Granada, Faculty of Sports Sciences, in collaboration with the PROFITH research group.

This operations manual includes a motor story, accompanying illustrations, and instructions for each test in the battery, such as handgrip strength, standing long jump, agility speed (4x10 m), one-leg balance, and the 20 m PREFIT shuttle run test. A graphic description accompanies each instruction to help understanding and show how each test relates to the motor story. Finally, annex documents are provided, including the recording sheet, the multiple lap sheet for the 20-meter shuttle run, and large illustrations for use during the battery testing.

Furthermore, the battery listed below is part of the project "Preschool Fit - Healthy and Smart: PREFIT-Chile Study Linking Physical Fitness to Non-Invasive Health-Related Markers and Executive Function," with code REDI 170474, funded by the 2017 competition for support in forming international networks for early-stage researchers by the National Commission for Scientific and Technological Research - CONICYT.

# Introducción

El presente documento tiene como finalidad ayudar y orientar la ejecución de la "Batería PREFIT: Evaluación en preescolares del fitness". Este se confecciona como una adaptación del protocolo original de la "Batería PREFIT: Evaluación del fitness en preescolares" de la Universidad de Granada, Facultad de Ciencias del Deporte en conjunto con el Grupo de investigación PROFITH.

El presente manual de operaciones se compone de un cuento motor, ilustraciones que acompañan dicho cuento y las instrucciones para cada uno de los test que componen la batería, tales como: fuerza de prensión manual, salto de longitud a pies juntos, velocidad de agilidad 4x10 m, equilibrio con una pierna y el test 20 m ida y vuelta PREFIT. A su vez, cada una de estas instrucciones está acompañada de una descripción gráfica para facilitar su comprensión y la respectiva asociación que tiene dicho test con el cuento motor. Finalmente, se proporcionan documentos anexos como la planilla de registros, la planilla múltiple de vueltas test de 20 metros de ida y vuelta PREFIT y las ilustraciones en tamaño grande para que puedan ser ocupados al momento de aplicar la batería.

Por otra parte, la batería presentada a continuación, se enmarca en el proyecto "Preschool Fit- healthy and smart: PREFIT-Chile Study Linking Physical Fitness to non invasive health-related markers and Executive Function" código REDI 170474, financiado por el concurso de apoyo a la formación de redes internacionales para investigadores(as) en etapa inicial 2017 de la Comisión Nacional de Investigación Científica y Tecnológica – CONICYT.

## 1. Motor Story

To enhance the interactivity of the assessment for children, the tests are introduced alongside a motor story. This story should be presented to the participants prior to the commencement of the tests, accompanied by illustrations. Subsequently, these illustrations will be associated with each test, enabling children to connect the previously introduced motor story with the actions required at each stage. At the conclusion of the operations manual, large-sized illustrations (Annex 3) are provided for use during the protocol implementation. Additionally, each test references the association with the story and the corresponding illustration utilized for the specific test.

**“Sam and Samanta in the Amazon Jungle”**

Hi there! I'm Sam, and she’s my sister Samanta. We're twins, and we absolutely love it because we're each other's playmates and partners in mischief. I adore adventures, and Samanta is passionate about exploring. Do you enjoy adventures and discovering new things? Well, today I want to share one of our many exciting adventures with you.

Yesterday, Mom took us to the zoo, a wonderful place filled with many animals, most of which come from the Amazon rainforest in Brazil. We haven't been there ourselves, but today we imagined it right in our backyard.

We stepped out onto the patio like we do every day, but this time, we wore some giant hats we found in Grandpa's old trunk. That was the beginning of our extraordinary adventure because the trunk's door was very heavy. I took a deep breath because it was super dusty, and Samanta pushed the door handle with all her might using one hand. And guess what? It opened! Yes, she did it! With our hats on, we became brave explorers, lost in the Amazon jungle that Mom told us about at the zoo.

Standing in the patio, we closed our eyes tightly and took the deepest breaths we could. When we opened them, the patio transformed into a wild jungle! Everything was lush, green, and humid. We could hear animal sounds all around us, and the only thing we had on us was our explorer hats.

We started strolling, excited because everything was so new and wondrous. As we explored, suddenly, we reached a dead end! We almost toppled over! In front of us was a deep ditch, so we gathered our courage and leapt as far as we could to the other side, trying to imitate a red-eyed frog we saw along the way. And Saz! We barely made it across, feeling like true adventurers. We were already feeling hungry, and a storm was starting to brew. Raindrops touched our faces, accompanied by a warm breeze that made everything feel cozy. I looked around and spotted a tree full of fruit, so I gently told Samanta to run as fast as she could to grab a few—one, two, three, four

of them—while we hurried back to hide under a big bush that we used as shelter (Agility speed 4x10 meters). Once we were safe from the rain, we began eating our tasty fruit, but suddenly, we heard a loud roar nearby that made us jump and cling to each other in surprise and a bit of fear! Samanta looked up at the tree where we got the fruit—can you guess what was up there? Just a big, black panther! A huge cat that loves to eat meat! We quickly decided it was best to run, and as we did, we saw many zebras gathered by a lagoon. I whispered to Samanta to hide among them when we came across some flamingos, and an idea popped into my head! We pretended to be flamingos! We stood on one leg and stayed perfectly still, like statues, hoping to go unnoticed (One-leg balance test). The panther, seeing all those zebra stripes and pink birds standing so still, got confused and decided to go back to its tree. It was unbelievable how fast we could run and hide, and we couldn't stop laughing about it for a long time. We kept walking through the animal-filled landscape until we reached the edge of the jungle again. The grass was so tall that we couldn't see where we were stepping. Suddenly, we fell into a very deep hole! To our surprise, it led to a bright, underground secret passage. Samanta thought it might be home to some giant moles, but we weren't sure… Then, an alarm went off, and a door further down the hallway swung open. That's when we realized we needed to reach each door before the alarm sounded again (20-meter shuttle run test). We were so caught up in our adventure and eager to reach each door in time that we didn't hear Mom calling us to take a bath. Just then, a funny red-bottomed monkey appeared and…welcomed us back! Just kidding! It was actually Mom, carrying us home to get clean. Well, that's all for now from Sam and Samanta—see you in our next adventure! Now it's your turn to create your own stories! Have lots of fun, and watch out for those speedy panthers—they run incredibly fast!

## 1. Cuento Motor

Con el objetivo de que la evaluación resulte interactiva para los niños y niñas, es que se proponen los test en conjunto con un cuento motor. Dicho cuento deberá ser expuesto a los evaluados antes de comenzar los test, acompañado por ilustraciones. Posteriormente dichas ilustraciones acompañaran cada una de las pruebas para que los niños y niñas asocien el cuento motor anteriormente expuesto a lo que deben realizar en el momento.

Al finalizar el manual de operaciones se encuentran las ilustraciones en tamaño grande (anexo 3) para disponer de ellas al momento de aplicar el protocolo, así mismo en cada uno de los test se menciona la asociación con el cuento y la ilustración correspondiente a la prueba.

**"Sam y Samanta en la selva amazónica"**

¡Hola! Mi nombre es Sam y ella es Samanta, nosotros somos mellizos y eso nos fascina porque somos compañeros de juegos y travesuras. ¡A mí me encantan las aventuras! Y a Samanta le encanta explorar, ¿Y a ustedes, les  agrada las aventuras y explorar? Bueno, hoy te contaré una de nuestras tantas aventuras que tenemos.

Ayer mamá nos llevó a conocer el zoológico, es un lugar donde se encuentran muchos animales de los cuales bastante de ellos provienen de la selva Amazónica de Brasil, ese lugar no lo conocemos, pero hoy lo imaginamos en el patio nuestra casa.

Salimos al patio como todos los días, pero esta vez nos pusimos unos sombreros enormes que encontramos en uno de los baúles del abuelo, ese fue el inicio de nuestra gran aventura ya que la puerta del cajón era muy pesada… Tuve que respirar profundo porque tenía mucho polvo, mientras que Samanta presionaba la manilla de la puerta con todas sus fuerzas con una sola mano **(Fuerza de prensión manual)** ¡Hasta que abrió! ¡Ja, si, lo logró! Con las gorras ya puestas podemos ser exploradores aventureros perdidos en esa selva amazónica que mamá nos nombró en el zoológico.

Al estar en el patio, cerramos nuestros ojos con mucha fuerza y respiramos lo más profundo que pudimos, al abrirlos ¡El patio era una selva! ¡Si! Todo era muy verde y húmedo, sentíamos ruidos de animales y lo único que llevábamos con nosotros era el sombrero de explorador.

Comenzamos a caminar muy lento, todo era nuevo para nosotros ¡Nos sentíamos muy emocionados! En eso que íbamos caminando ¡Oh! ¡Se nos acabó el camino! ¡Casi caímos! Frente de nosotros había una zanja muy profunda por lo cual tuvimos que usar toda nuestra fuerza para dar un salto a pies juntos lo más lejos posible para llegar al otro lado **(Salto de longitud a pies juntos)**; imitamos a una rana de ojos rojos que vimos en el camino y ¡Saz! Logramos cruzar por poco.

Ya teníamos hambre y comenzaba una tormenta, sentíamos gotas de lluvia sobre nuestras caras junto con la brisa de un viento cálido, miré a mi alrededor y vi un árbol que tenía mucha fruta así que le dije a Samanta que corriéramos a toda velocidad para tomar una, dos, tres, cuatro de ellas mientras

corríamos de vuelta a dejarlas debajo de un arbusto muy grande que usamos de refugio **(Velocidad de agilidad 4x10metros)**. Una vez ya protegidos de la lluvia empezamos a comer nuestra fruta, pero escuchamos un rugido muy cerca de nosotros que la verdad ¡Nos hizo saltar y abrazarnos de miedo! Samanta miró el árbol de donde sacamos la fruta y ¡No saben que había sobre él! ¡Una pantera! Una especie de gato negro enorme ¡Que se alimenta de carne! Comenzamos a correr y vimos muchas cebras que estaban juntas a la orilla de una laguna, le dije a Samanta que nos escondiéramos entre ellas cuando nos topamos con unos flamencos que me dieron una brillante idea ¡Las imitamos! Nos paramos sobre solo uno de nuestros pies y nos quedamos como una estatua por el mayor tiempo posible **(Test de equilibrio con una pierna)**. La pantera, al ver tantas rayas de cebras juntas y unos pájaros rosados altos, muy quietos, se mareó y se devolvió a su árbol. Nunca pensamos correr y escondernos tan rápido como lo hicimos, lo que nos hizo reír por mucho rato.

Seguimos nuestro camino entre los animales hasta llegar nuevamente a la orilla de la selva. El pasto estaba tan largo que no nos dejaba ver por donde caminábamos. En eso que ibas peleando con el pasto. ¡Caímos a un agujero muy hondo! Descubrimos que nos llevaba a ¡un pasadizo secreto muy iluminado bajo tierra! Samanta creía que era la casa de unos topos enormes, pero no lo sabemos… Sonó una alarma y se abrió una puerta más adelante del pasillo largo; fue así como entendimos que debíamos llegar a cada puerta que se nos aparecía en el camino antes de que volviera a sonar la alarma **(Test de 20 metros de ida y vuelta)**.

Estábamos tan concentrados en la aventura y en llegar a cada puerta antes del sonido que no oímos a mamá llamando que debíamos bañarnos, así que un mono poto colorado nos tomó por sorpresa y ¡Nos llevó! ¡Era broma! Solo era mamá quien nos llevaba en brazo de vuelta a casa para poder bañarnos.

Bueno, somos Sam y Samanta, ¡Nos vemos en nuestra próxima aventura, pues ahora les toca a ustedes vivir la suya! Que se diviertan y… ¡Cuidado con las panteras corren muy rápido!

## 2. TEST

### 2.1 Capacidad músculo- esquelética

#### 2.1.1 Fuerza de prensión manual

| | |
|---|---|
| **Propósito** | Medir la fuerza isométrica del tren superior. |
| **Relación con salud** | La fuerza muscular está inversamente asociada con factores de riesgo de enfermedad cardiovascular establecidos y emergentes, dolor de espalda y con la densidad y contenido mineral óseo en niños y adolescentes. |
| **Material** | Dinamómetro analógico con agarre ajustable (TKK 5001 Grip A, modelo analógico, rango de medición 0-100; Takey, Tokio Japan). |
| **Ejecución** | **Descripción:** El niño/a apretará el dinamómetro poco a poco y de forma continua durante al menos 2 segundos, realizando el test en dos ocasiones (alternativamente con las dos manos) con el ajuste óptimo de agarre en 4.0 cm y permitiendo un breve descanso entre las medidas. Para cada medida, se elegirá al azar estar en toda su extensión y se evitará el contacto del dinamómetro con cualquier parte del cuerpo, salvo con la mano que se está midiendo.<br>**Instrucciones para el evaluado:** Sostiene el dinamómetro con una mano. Apretarás con la mayor fuerza posible procurando que el dinamómetro no toque tu cuerpo. Apretarás gradualmente y de forma continua durante al menos 2 segundos.<br>**Relación con el cuento:** Mientras que Samanta presionaba la manilla de la puerta con todas sus fuerzas con una sola mano.<br>**Práctica y número de ensayos:** El examinador mostrará la forma correcta de ejecución. El test se realizará dos veces y el mejor resultado será registrado. |
| **Medida** | La duración máxima de la prueba será de 3-5 segundos. La precisión de la medida es de 0,5 kg. Durante la prueba, el brazo y la mano que sostiene el dinamómetro no deberán tocar el cuerpo. El instrumento se mantendrá en línea con el antebrazo. Después de un breve descanso, se realizará un segundo intento. El indicador se pondrá a cero después del primer intento. |
| **Puntuación** | Para cada mano, se registra el mejor intento (en kilogramos, precisión 0,5 kg).<br>*Ejemplo:* un resultado de 4 kg se registra 4,0. |
| **Video** | https://youtu.be/XiqsEjB2Hnc |

Descripción grafica del test fuerza de prensión manual:

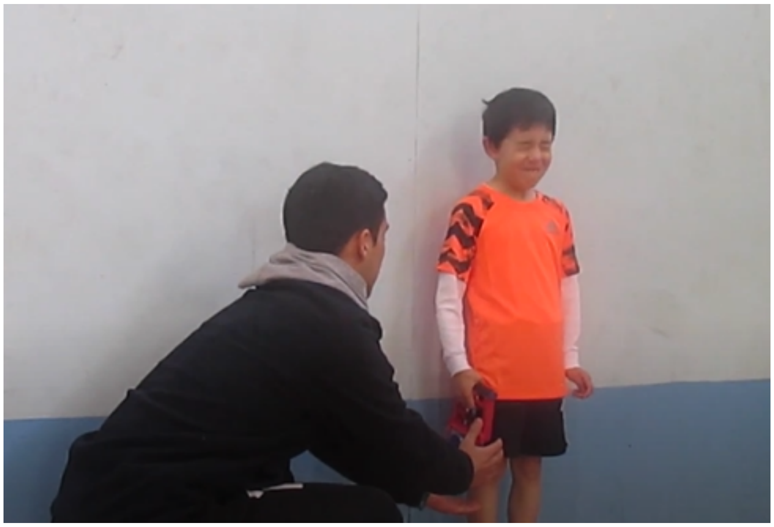

Ilustración del cuento relacionada con el test:

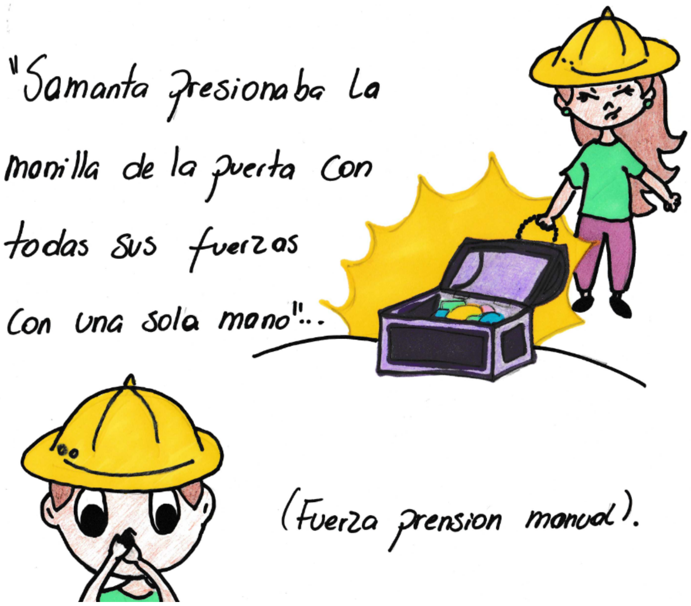

### 2.1.2 Salto de longitud a pies juntos

| | |
|---|---|
| **Propósito** | Medir la fuerza explosiva del tren inferior. |
| **Relación con salud** | La fuerza muscular está inversamente asociada con factores de riesgo de enfermedad cardiovascular, dolor de espalda y con la densidad y contenido mineral óseo. Mejoras de la fuerza muscular de la infancia a la adolescencia se asocian inversamente con los cambios en la adiposidad total. |
| **Material** | Superficie dura no deslizante, stick o pica plana, una cinta métrica, cinta adhesiva y conos (no obligatoria). |
| **Ejecución** | **Descripción:** El niño/a se colocará de pie tras la línea de salto, y con una separación de pies igual a la anchura de sus hombros. Desde esa posición, doblará las rodillas con los brazos delante del cuerpo y paralelo al suelo, balanceará los brazos, empujará con fuerza y saltará lo más lejos posible. Tomará contacto con el suelo con los dos pies simultáneamente y en posición vertical.<br>**Instrucciones para el evaluado:** Coloca los pies en la línea de salida. Saltarás lo más lejos posible con los dos pies juntos. Te quedarás quieto/a cuando toques el suelo.<br>**Relación con el cuento:** Frente de nosotros había una zanja muy profunda por lo cual tuvimos que usar toda nuestra fuerza para dar un salto a pies juntos lo más lejos posible para llegar al otro lado.<br>**Práctica y número de ensayos:** El examinador mostrará la forma correcta de ejecución. El test se realizará tres veces y el mejor resultado será registrado. |
| **Medida** | Líneas horizontales se dibujarán en la zona de caída o aterrizaje a 10 cm de distancia, a partir de 1 m de la línea de despegue. Una cinta métrica perpendicular a estas líneas dará las medidas exactas. El examinador estará junto a la cinta métrica y registrará la distancia saltada por el niño/a. La distancia saltada se medirá desde la línea de despegue hasta la parte posterior del talón más cercano a dicha línea. Se permitirá un nuevo intento si el niño/a cae hacia atrás o hace contacto con la superficie con otra parte del cuerpo. |
| **Puntuación** | Para cada mano, se registra el mejor intento (en kilogramos, precisión 0,5 kg).<br>El resultado se registra en cm.<br>Ejemplo: un salto de 1 m 06 cm, se registra 106. |
| **Video** | https://youtu.be/XiqsEjB2Hnc |

Descripción grafica del test salto de longitud a pies juntos:

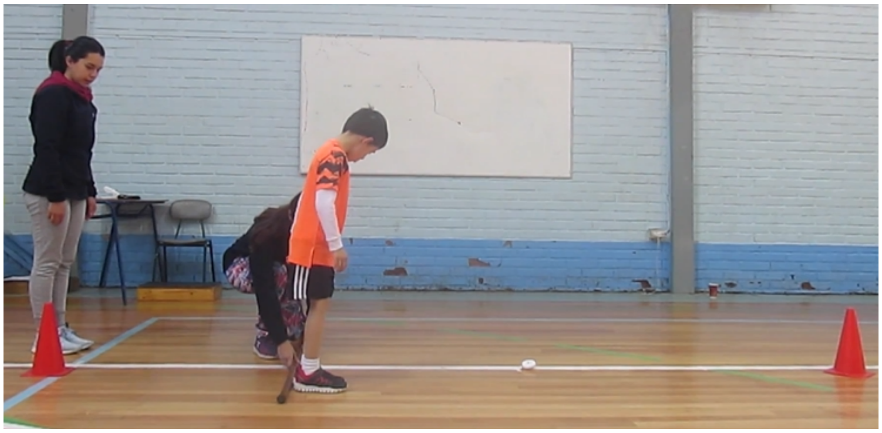

Ilustración del cuento relacionada con el test:

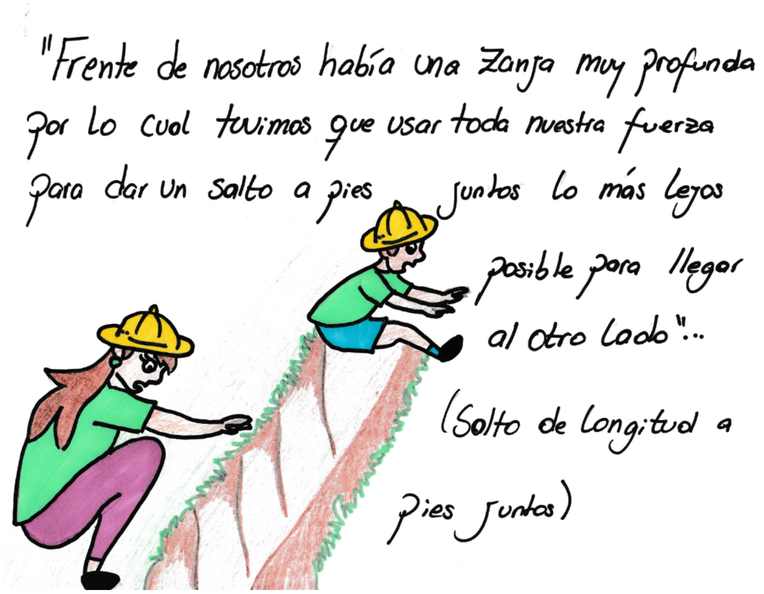

### 2.2 Capacidad motora

#### 2.2.1 Velocidad de agilidad 4x 10m

| | |
|---|---|
| **Propósito** | Medir la velocidad de movimiento, agilidad y coordinación. |
| **Relación con salud** | Mejoras en la velocidad/agilidad parecen tener un efecto positivo sobre la salud de los huesos. |
| **Material** | Superficie limpia y no deslizante, cronómetro, 4 conos y cinta adhesiva. |
| **Ejecución** | **Descripción:** Test de correr y girar a la máxima velocidad (4x10 m). Dos líneas paralelas se dibujarán en el suelo (con cintas) a 10 metros de distancia, situándose un examinador en cada extremo. Cuando se indique la salida, el niño/a correrá lo más rápido posible a la otra línea, le chocará la mano al examinador 1 (tipo "choca esos 5") y volverá a la línea de salida, cruzando ambas líneas con los dos pies y *chocándole* la mano al examinador 2. Seguidamente, de nuevo, irá corriendo lo más rápido posible a la línea opuesta, le *chocará* la mano al examinador 1 y volverá corriendo a la línea de salida inicial donde le *chocará* la mano al examinador 2.<br>**Instrucciones para el evaluado:** Prepárate detrás de la línea de salida. Cuando se indique el inicio, correrás tan rápido como sea posible a la otra línea, *chocarás* la mano del examinador 1 (tipo "choca esos 5") y volverás a la línea de salida. Luego, volverás corriendo lo más rápido posible a la línea opuesta y chocarás la mano del examinador 2. Asegúrate de pasar ambas líneas con los dos pies. Por último, volverás de nuevo a la línea de salida sin reducir tu velocidad hasta haberla cruzado.<br>**Relación con el cuento:** Le dije a Samanta que corriéramos a toda velocidad para tomar una, dos, tres, cuatro de ellas mientras corríamos de vuelta a dejarlas debajo de un arbusto muy grande que usamos de refugio.<br>**Práctica y número de ensayos:** El examinador mostrará la forma correcta de ejecución. El test se realizará dos veces y el mejor resultado será registrado. |
| **Medida** | Asegúrese que los dos pies cruzan la línea cada vez, que el niño realiza el recorrido requerido y que los giros lo realiza lo más rápido posible. Enumere en voz alta los ciclos completados. El test finalizará cuando el niño/a cruza la línea de llegada (en un primer momento línea de salida) con un pie. El niño/a no deberá deslizarse |

| | |
|---|---|
| | o resbalarse durante la prueba, por lo que es necesaria una superficie antideslizante. |
| **Puntuación** | El resultado se registra en segundos con un decimal.<br>Ejemplo: un tiempo de 21,6 segundos se anotará como 21,6. |
| **Video** | [https://youtu.be/XiqsEjB2Hnc](https://youtu.be/XiqsEjB2Hnc) |

---

Descripción grafica del test velocidad de agilidad 4x10m:

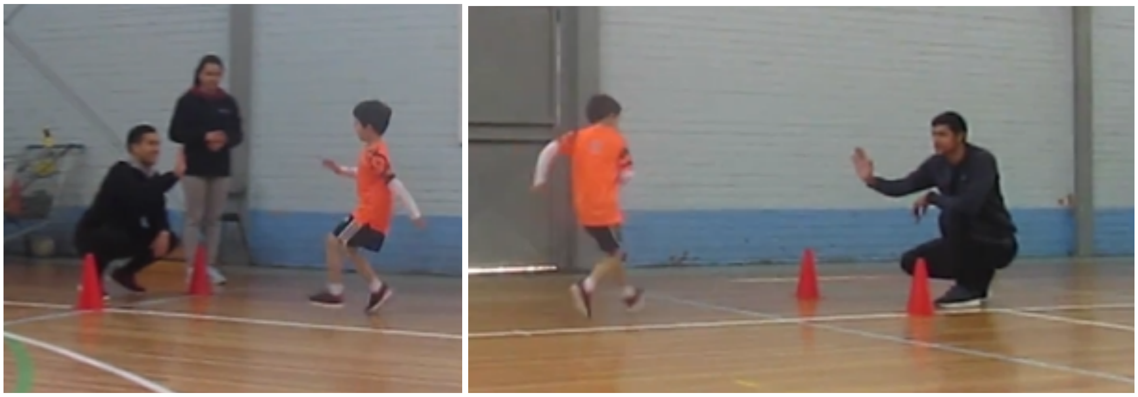

Ilustración del cuento relacionada con el test:

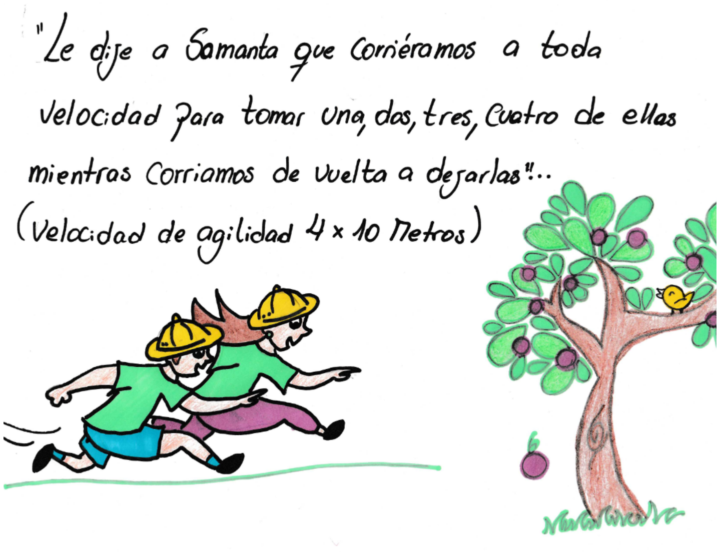

### 2.3 Equilibrio

#### 2.3.1 Test de equilibrio con una pierna

| | |
|---|---|
| **Propósito** | Medir el equilibrio estático. |
| **Relación con salud** | Niveles bajos de equilibrio a edades tempranas pueden ser indicadores de algún problema o patología del sistema neuro-muscular. |
| **Material** | Superficie dura no deslizante y cronómetro. |
| **Ejecución** | **Descripción:** El niño/a se situará de forma estática sobre el suelo y con una pierna flexionada. Se efectuará un intento con cada pierna, anotando el tiempo que se consiga mantener en esa posición.<br>Este test consiste en mantener el equilibrio durante el mayor tiempo posible. El tiempo estará controlado por un cronómetro. Se situará de forma estática, teniendo la pierna de apoyo sobre el suelo y la otra flexionada. Efectuará un intento con cada pierna. Utilizará los brazos para equilibrarse cuando sea necesario. Su objetivo será mantener el equilibrio en la posición establecida el mayor tiempo que le sea posible. Se activará el cronómetro cuando la pierna libre deje el suelo. El test finalizará cuando no pueda mantener la posición requerida, es decir, mueva el pie de apoyo, talón o punta, de la posición original; toque el suelo con el pie libre o cuelgue o apoye la pierna libre en la de apoyo. La prueba evalúa el equilibrio estático.<br>**Instrucciones para el evaluado:** Apoya una pierna sobre el suelo y la otra la flexionas durante el mayor tiempo posible. Utiliza los brazos para equilibrarte siempre que sea necesario. Terminarás el test cuando la pierna que está flexionada toque el suelo. Realizarás esta prueba dos veces, una con cada pierna.<br>**Relación con el cuento:** Nos topamos con unos flamencos que me dieron una brillante idea ¡Las imitamos! Nos paramos sobre solo uno de nuestros pies y nos quedamos como una estatua por el mayor tiempo posible.<br>**Práctica y número de ensayos:** Previo al desarrollo de la prueba, realizará un ensayo con cada pierna donde el examinador le ayudará al niño/a a adoptar una postura equilibrada, sosteniéndole una o dos manos si fuese necesario. El test lo realizará una vez con cada pierna y registrará la media. |
| **Medida** | Asegúrese que la posición inicial es la correcta y que la pierna se encuentra flexionada. Anote el tiempo que consigue mantener esta |

| | |
|---|---|
| | posición en cada intento. Es necesario evitar elementos distractores que puedan influir en el resultado de la prueba. |
| **Puntuación** | El resultado se registra en segundos con un decimal.<br>Ejemplo: una duración de 15,3 segundos. |
| **Video** | https://youtu.be/XiqsEjB2Hnc |

---

Descripción grafica del test equilibrio con una pierna:

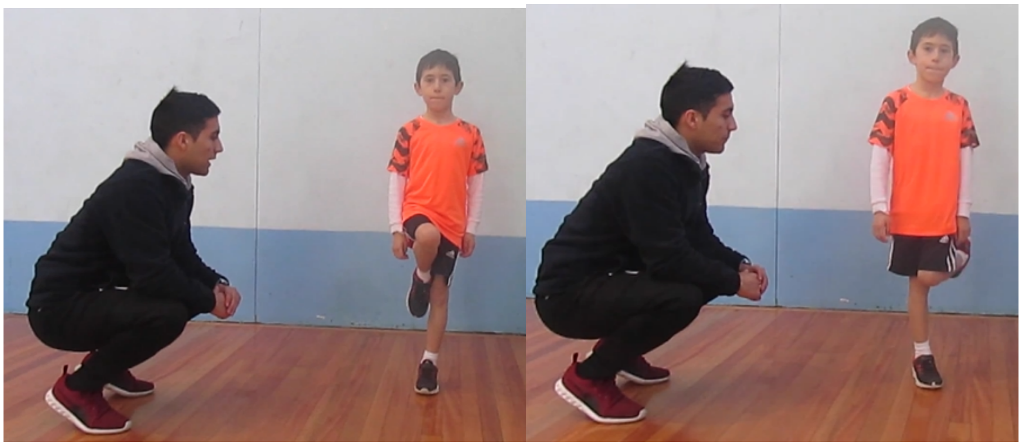

Ilustración del cuento relacionada con el test:

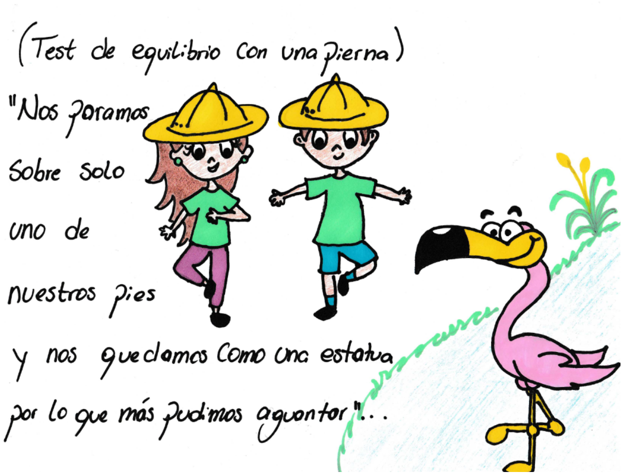

## 2.4 Capacidad cardiorrespiratoria

### 2.4.1 Test de 20m de ida y vuelta PREFIT

| | |
|---|---|
| **Propósito** | Medir la capacidad cardiorrespiratoria |
| **Relación con salud** | Niveles altos de capacidad cardiorrespiratoria durante la niñez y la adolescencia están asociados con una salud cardiovascular actual y futura más saludable. |
| **Material** | Un gimnasio o un espacio lo suficientemente grande para marcar una distancia de 20 metros, 4 conos, cinta métrica, CD con el protocolo del test y un reproductor de CD. |
| **Ejecución** | **Descripción:** El niño/a se desplazará de una línea a otra situadas a 20 metros de distancia y haciendo el cambio de sentido al ritmo indicado por una señal sonora que irá acelerándose progresivamente. Se recomienda que al menos un examinador realice la prueba con los niños. Idealmente, el test requiere de 2 personas corriendo con los niños, uno por delante y otro por detrás de ellos, formando una franja imaginaria en movimiento que les ayuda a mantener la velocidad adecuada. La velocidad inicial de la señal es de 6,5 km/h, y se incrementará en 0,5 km/h/min (1 minuto es igual a 1 palier/estadío). Esta es la mayor adaptación realizada del test original (agregar citas).<br>La prueba terminará cuando el niño/a no sea capaz de llegar por segunda vez consecutiva a una de las líneas con la señal de audio. De lo contrario, la prueba terminará cuando el niño se detiene debido a la fatiga.<br>**Instrucciones para el evaluado:** Este test consiste en ir y volver corriendo. La velocidad será controlada por medio de un CD que emite sonidos. Adecuarás tu ritmo al ritmo de los examinadores con el fin de estar en uno de los extremos de la pista cuando el reproductor emita un sonido. Tocarás la línea al final de la pista con el pie y correrás en la dirección opuesta. Al principio, la velocidad será baja, pero se incrementará lentamente y de manera constante cada minuto. Tu objetivo en la prueba será seguir el ritmo marcado el mayor tiempo que te sea posible. Por lo tanto, deberás detenerte cuando el examinador te lo diga o no puedas mantener el ritmo establecido.<br>**Relación con el cuento:** Sonó una alarma y se abrió una puerta más adelante del pasillo largo, fue así como entendimos que |

| | |
|---|---|
| | debíamos llegar a cada puerta que se nos aparecía en el camino antes que volviera a sonar la alarma. **Práctica y número de ensayos:** Esta prueba se realizará una vez. |
| **Medida** | Seleccione el sitio de prueba, preferentemente que sea un gimnasio de 25 m de largo o más. Permita un espacio de al menos un metro en cada extremo de la pista. Cuanto más amplia sea la superficie utilizada, mayor el número de niños que podrán realizar simultáneamente la prueba: se recomienda un metro para cada niño/a. La superficie deberá ser uniforme, aunque el material del que está hecho no es especialmente importante. Los dos extremos de la pista de 20 metros deberán estar claramente marcados (conos). Compruebe el funcionamiento y el sonido del reproductor de CD. Asegúrese de que el dispositivo es lo suficientemente potente como para evaluar a un grupo. Escuche el contenido del CD. Anote los números del contador de tiempo del reproductor de CD con el fin de poder localizar las secciones clave de la pista rápidamente. |
| **Puntuación** | Una vez que el niño/a se detiene, un examinador externo registrará el número de vueltas completadas (Anexo 2). A posteriori, la conversión del número de vueltas a estadíos se puede realizar observando la tabla 1 de este manual. Ejemplo: una puntuación de 5 vueltas correspondería al estadío 1. Si es necesario una mayor precisión (por ejemplo, estudios de intervención con el objetivo de detectar pequeños cambios), se recomienda registrar el número de vueltas alcanzadas en la prueba, en lugar de estadíos completados. |
| **Video** | https://youtu.be/XiqsEjB2Hnc |
| **Audio** | https://drive.google.com/file/d/1y1kWOgYjkswHM8QXug7bOow4JlSc9Ztd/view?usp=sharing |

Descripción grafica del test 20m ida y vuelta PREFIT:

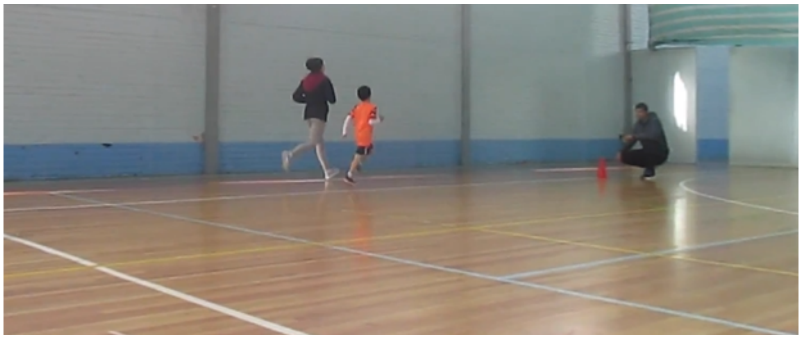

Ilustración del cuento relacionada con el test:

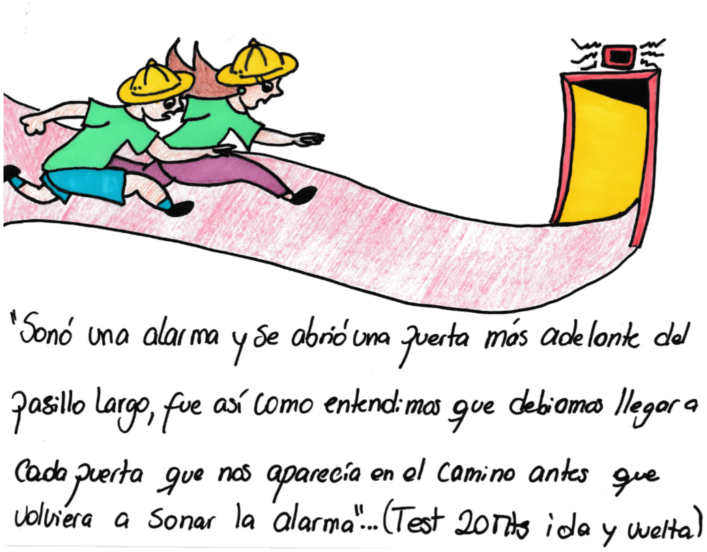

**Tabla 1.** Estadíos, Velocidad de carrera (km/h) y número de vueltas en la adaptación del test de 20 m de ida y vuelta PREFIT.

| **Estadíos** | **Velocidad (km/h)** | **Vueltas** | **Vueltas totales** |
|---|---|---|---|
| **0,5** | 6,5 | 3 | 3 |
| **1** | 6,5 | 2 | 5 |
| **1,5** | 7,0 | 3 | 8 |
| **2** | 7,0 | 3 | 11 |
| **2,5** | 7,5 | 3 | 14 |
| **3** | 7,5 | 3 | 17 |
| **3,5** | 8,0 | 3 | 20 |
| **4** | 8,0 | 4 | 24 |
| **4,5** | 8,5 | 4 | 27 |
| **5** | 8,5 | 3 | 31 |
| **5,5** | 9,0 | 4 | 35 |
| **6** | 9,0 | 4 | 39 |
| **6,5** | 9,5 | 4 | 43 |
| **7** | 9,5 | 4 | 47 |
| **7,5** | 10,0 | 4 | 51 |
| **8** | 10,0 | 4 | 55 |
| **8,5** | 10,5 | 5 | 59 |
| **9** | 10,5 | 4 | 64 |
| **9,5** | 11,0 | 5 | 68 |
| **10** | 11,0 | 4 | 73 |
| **10,5** | 11,5 | 5 | 78 |
| **11** | 11,5 | 5 | 83 |
| **11,5** | 12,0 | 5 | 88 |
| **12** | 12,0 | 5 | 93 |
| **12,5** | 12,5 | 5 | 98 |
| **13** | 12,5 | 5 | 103 |

### 2.5 Consideraciones prácticas

La siguiente tabla destaca las recomendaciones prácticas para cada uno de los test propuestos de la batería PREFIT en niños de 3 a 5 años.

**Tabla 2.** Consideraciones prácticas para el desarrollo de la batería PREFIT, evaluación del **FIT**ness en **PRE**escolares de 3 a 5 años.

| Test de condición física | Consideraciones prácticas/ recomendaciones |
|---|---|
| Todos los test | - Es extremadamente importante que se esté continuamente motivando y animando a los niños durante la realización de todos los tests. Se recomienda hacer la evaluación como un juego, siendo cada test como pruebas de aventura. Una buena estrategia es contar un cuento en el que los niños tienen que ayudar al protagonista a pasar diversas aventuras (para contar el cuento los niños pueden estar sentados en círculo y el examinador en medio de éste).<br>- En cada estación el examinador le deberá recordar al niño en qué aventura se encuentra respecto al cuento motor.<br>- Se recomienda un mínimo de 2 examinadores, aunque idealmente se aconseja estar 5 examinadores<br>- En cuanto a la organización y estructura de la evaluación, los niños estarán en la clase y uno de los examinadores va a recogerlos en grupos de 8. Los 8 niños están organizados por parejas, y cada pareja se dirigirá a una de las 4 estaciones (1=antropometría, 2=fuerza, 3=salto, 4= velocidad-agilidad). El test de 20 m ida y vuelta PREFIT se realizará al final de la sesión de evaluación debido a que es un test máximo y los niños acaban cansados/fatigados.<br>- Los tests se realizan en la misma zona, aunque idealmente la parte de antropometría se recomienda hacerla en una habitación aparte.<br>- Se recomienda realizar un ejemplo antes del test.<br>- Se registran todas los resultados de todos los intentos realizados, aunque la mejor puntuación se utilizará para el análisis (excepto para antropometría que se utilizará la puntuación media). |
| Test de fuerza de prensión manual | - Recomendamos utilizar la versión analógica del TKK (modelo 5001) debido a que la digital (modelo 5401) mide de 5kg a 100kg, y nuestra experiencia nos ha |

| | |
|---|---|
| | demostrado que algunos niños en edad preescolar tienen un rendimiento en la prueba de fuerza inferior a 5kg. (véase artículo metodológico sobre la fiabilidad y validez de estos dinamómetros*)<br>- En un estudio previo, observamos que el agarre óptimo para niños en edad preescolar fue 4.0 cm*<br>- Los niños tienden a coger dinamómetro con las dos manos y apretar con ellas simultáneamente. Esto es algo que hay que corregir la mayoría de las veces. |
| Test de salto a pies juntos | - Los niños más pequeños (especialmente los de 3 años) tienen problemas para saltar con los pies juntos y caer al suelo sin caerse. Por lo tanto, se recomienda realizar uno o dos intentos de familiarización.<br>- También se recomienda realizar 3 intentos en lugar de 2 en comparación con el resto de los tests.<br>- Se recomienda dibujar las huellas de los pies en el suelo (con tiza) para guiar y hacer saber al niño cual es la línea de salida. |
| Test de velocidad-agilidad 4x 10m | - Para hacer el test más simple, se recomienda no utilizar esponjas para ser intercambiadas al cruzar las líneas de 10m de distancia (4 veces x 10m), como se hace en la batería ALPHA para niños y adolescentes. Para una mejor adecuación del test y consecución de los objetivos del mismo, se recomienda que en cada uno de los extremos haya un examinador. Así, el niño tendrá que chocar la mano al examinador y girarse para seguir con el test. Esto asegura que el niño/a hace el recorrido completo e incluye un componente motivacional adicional, chocar la mano<br>- El examinador podrá transmitirle las instrucciones durante la carrera. Ejemplo: “Ahora me chocas la mano y te das la vuelta rápidamente”. |
| Test de equilibrio a una pierna | - Hay muchas versiones de este test (por ejemplo, ojos abiertos frente a ojos cerrados, de pie en el suelo o sobre una barra), nosotros recomendamos usar el más simple cuando lo aplicamos con preescolares. El niño se sitúa de pie sobre un suelo no deslizante con los ojos abiertos. Se anota el tiempo (segundos con un decimal) que la persona es capaz de mantenerse en esa posición. El test se realiza una vez con cada pierna. |
| Test de 20 metros de ida y vuelta PREFIT | - Los niños/as en edad preescolar tienen problemas para mantener el ritmo adecuado de la prueba. Por lo tanto, se recomienda que uno o dos examinadores corran con |

| | |
|---|---|
| | ellos. De haber suficientes evaluadores, se recomienda que 2 corran con ellos, uno por delante y otro por detrás, creando un espacio imaginario en el que los niños van corriendo a la velocidad adecuada.<br>- Esta prueba se recomienda que se realice por grupos de 4- 8 niños (el número de niños variará dependiendo del número de examinadores). En niños de 3 años, se aconseja que los grupos sean reducidos 4-5 personas. Si solo hay un examinador, no se recomienda realizar el test con más de 3 -4 niños ( para aquellos de 4 y 5 años) y de 1- 2 (para niños de 3 años)<br>- En cuanto a la puntuación, en este test se registra el número de vueltas (1 vuelta= 20 metros) en lugar de los estadíos como se hace con niños mayores (la conversión de vueltas a estadíos se puede hacer a posteriori si fuese necesario). Esto hará la prueba más precisa y discriminante. |

## 3. ANEXOS

### ANEXO 1: Hoja de registro

# Batería PREFIT: Evaluación de la condición física en Preescolares

## MEDIDAS

**Nombre y Apellidos: ________________________________________________ Sexo: V/M**

**Colegio:______________________________ Curso:________**

| **TEST** Fecha: | | **RETEST** Fecha: | |
|---|---|---|---|
| Prensión manual-mano derecha (kg) | | Prensión manual-mano derecha (kg) | |
| Prensión manual-mano izquierda (kg) | | Prensión manual-mano izquierda (kg) | |
| Prensión manual-mano derecha (kg) | | Prensión manual-mano derecha (kg) | |
| Prensión manual-mano izquierda (kg) | | Prensión manual-mano izquierda (kg) | |
| Salto de longitud (cm) | | Salto de longitud (cm) | |
| Salto de longitud (cm) | | Salto de longitud (cm) | |
| Salto de longitud (cm) | | Salto de longitud (cm) | |
| Test 4x10(m) Vueltas | | Test 4x10(m) Vueltas | |
| Segundos | | Segundos | |
| Equilibrio pierna derecha | | Equilibrio pierna derecha | |
| Equilibrio pierna izquierda | | Equilibrio pierna izquierda | |
| Test 20 (m) Vueltas | | Test 20 (m) Vueltas | |

**Notas:** (ej. Razones de exclusión, problemas durante la realización de los test)

**Nombre/s examindador/a/es/as:**

____________________________________________________________.

**ANEXO 2: Planilla múltiple de vueltas test 20m ida y vuelta Prefit**

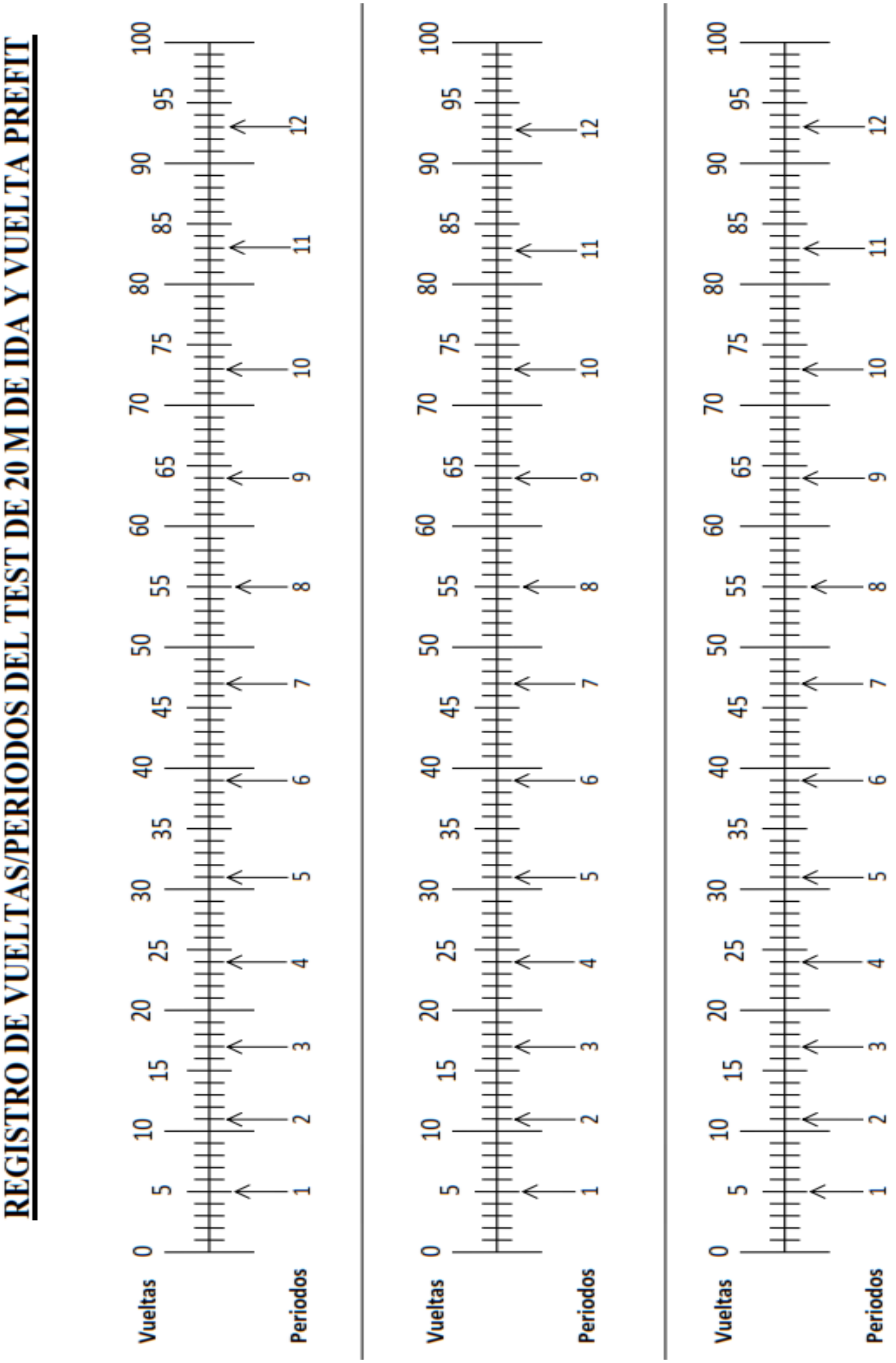

**ANEXO 3: Ilustraciones cuento motor.**

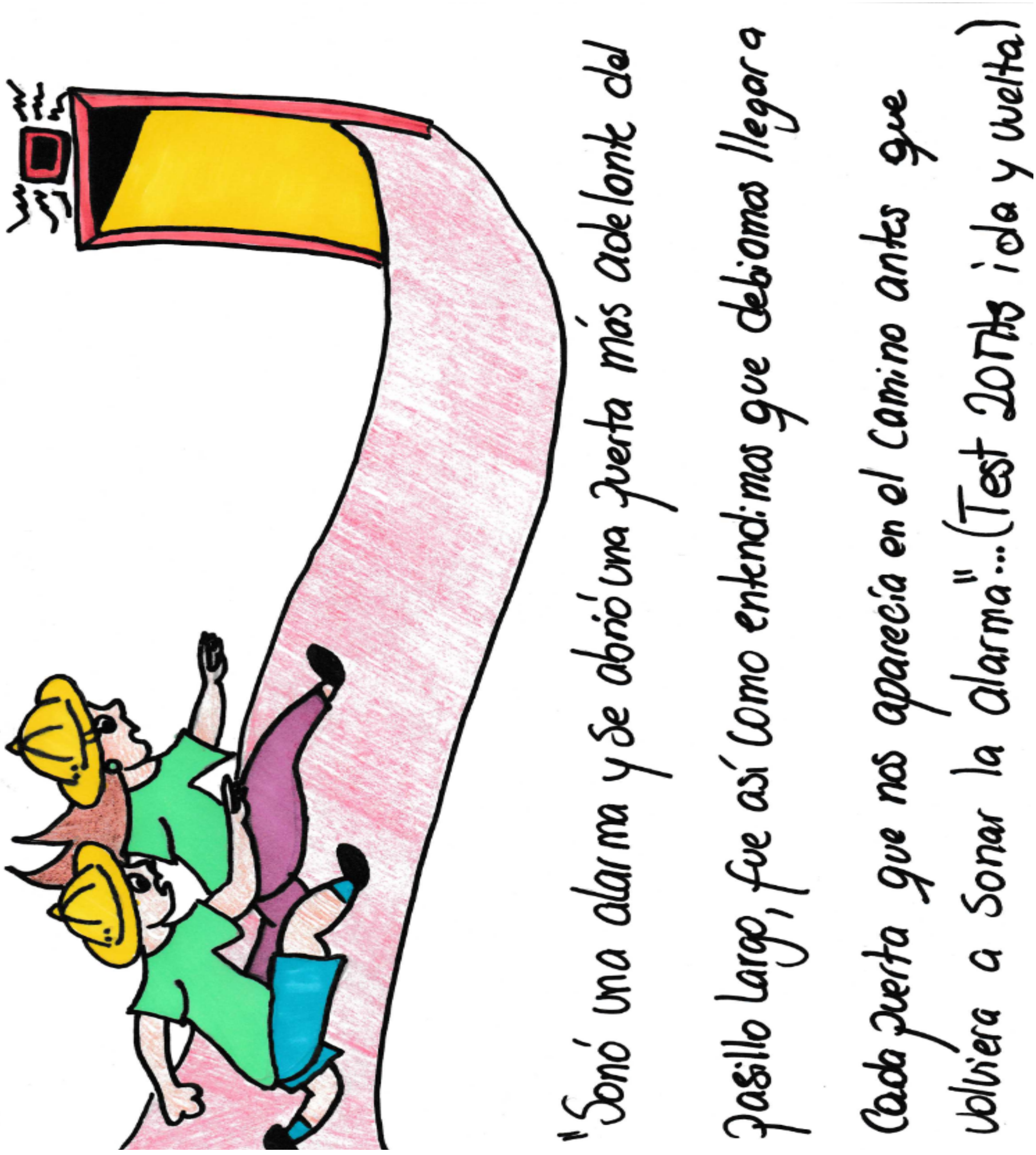

"Samanta presionaba la manilla de la puerta con todas sus fuerzas con una sola mano"...

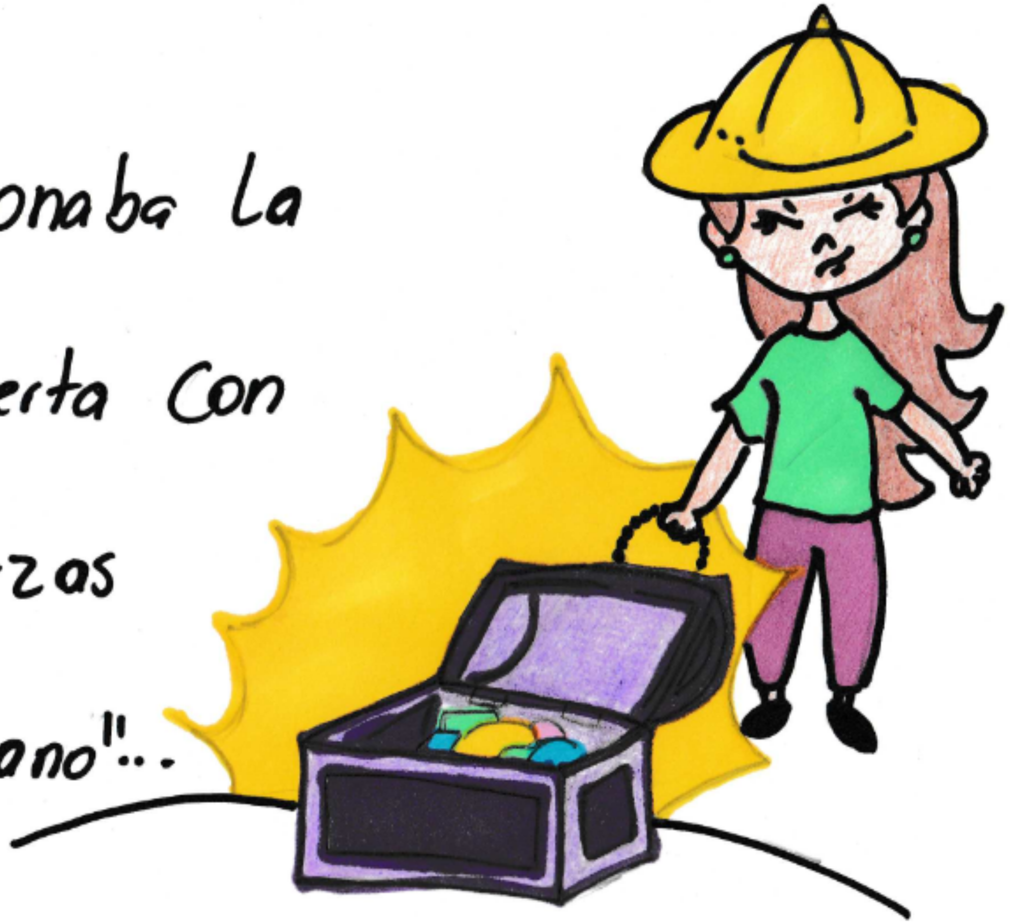

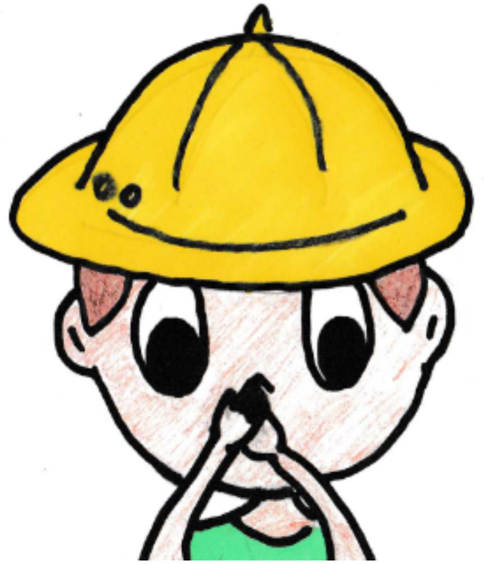

(Fuerza prension manual).

(Test de equilibrio con una pierna)

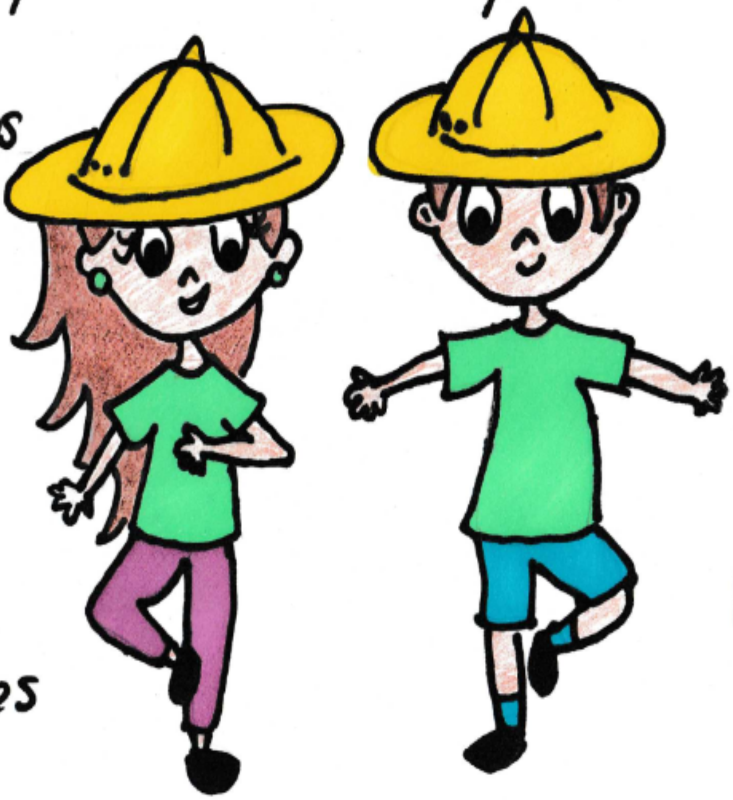

"Nos paramos

sobre solo

uno de

nuestros pies

y nos quedamos como una estatua

por lo que más pudimos aguantar"...

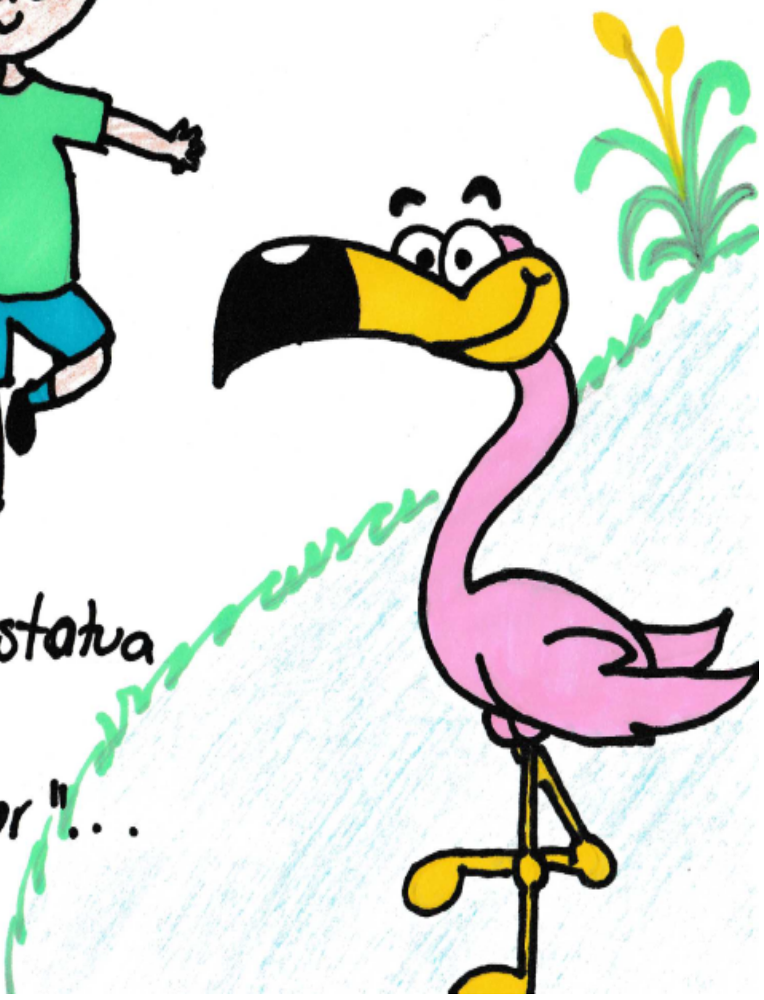

"Le dije a Samanta que corriéramos a toda velocidad para tomar una, dos, tres, cuatro de ellas mientras corriamos de vuelta a dejarlas"...
(Velocidad de agilidad 4 x 10 Metros)

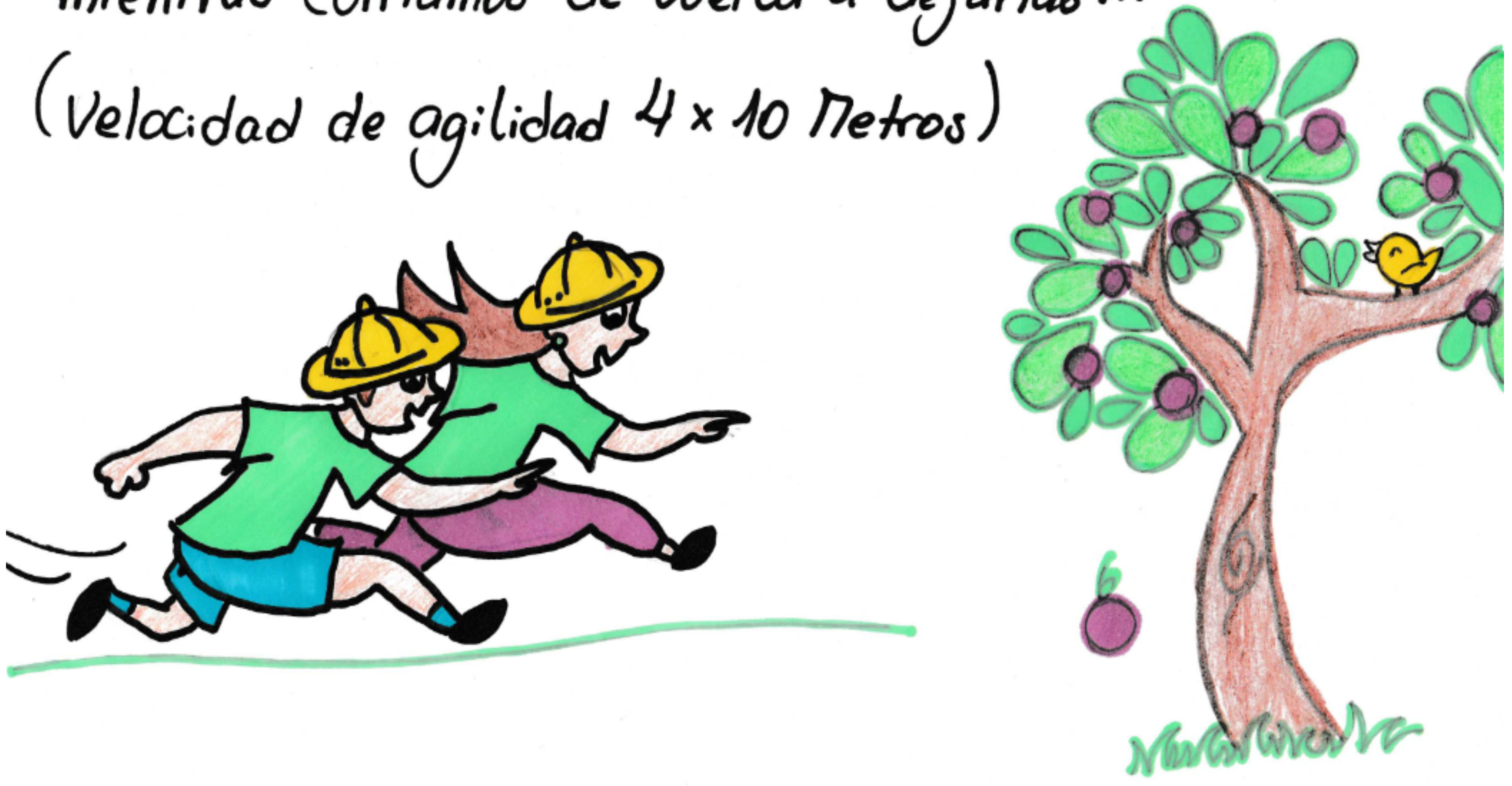

"Frente de nosotros había una Zanja muy profunda por lo cual tuvimos que usar toda nuestra fuerza para dar un salto a pies juntos lo más lejos posible para llegar al otro lado"...

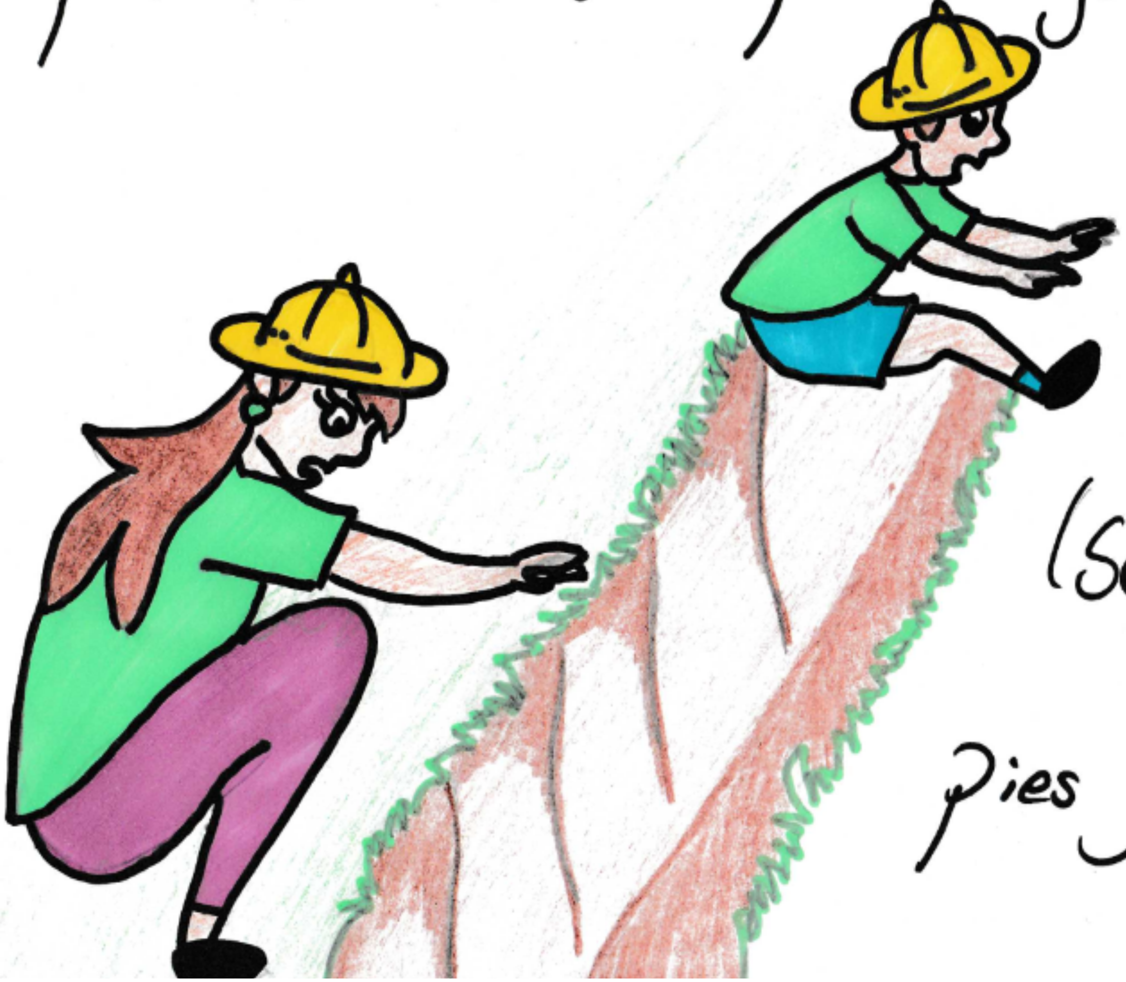

(Salto de longitud a pies juntos)
