# Supplementary material for: Reliability of the PREFIT fitness-test battery in Chilean preschoolers
Source: Front Pediatr. 2026 Jan 12;13:1654731. doi: 10.3389/fped.2025.1654731 (PMC12833291; doi:10.3389/fped.2025.1654731)
Supplement: Supplementary file 2 [file Supplementaryfile2.docx]

**Fig. 1 Reliability Assessment Process: Flowchart**

Analysed (n= 171)

Lost to follow-up, not attending on the day of the assessment

(n= 34)

Not attending on the day of the assessment

(n= 44)

## Enrollment

## Analysis

## Re-test

## Test

Assessed for eligibility

(n= 249)

Table 1. Test and retest and feasibility and reliability of PREFIT physical fitness tests.

| Physical Fitness test | ICC  Preschooler | 95% CI | ICC  School | SEM | MDC95 | CV% |
| --- | --- | --- | --- | --- | --- | --- |
| PREFIT 20 m shuttle run (laps) a | 0.6678 | 0.582 – 0.744 | 0.0577 | 5.334 | 14.785 | 3.054 |
| Handgrip strength (kg) a | 0.8590 | 0.809 – 0.897 | 0.1496 | 0.849 | 2.353 | 1.325 |
| Standing long jump (cm) a | 0.5824 | 0.487 – 0.672 | 0.0322 | 12.434 | 34.465 | 1.185 |
| 4 × 10 m shuttle run(s) a | 0.7785 | 0.710 – 0.835 | 0.1000 | 0.892 | 2.471 | 0.537 |
| One-leg stance (s) a | 0.4944 | 0.355 – 0.634 | 0.1785 | 6.674 | 18.500 | 2.673 |

ICC = Intraclass correlation; SEM = Standard Error of Measurement; MDC95 = Minimal Detectable Change at 95% confidence; CV% = percentage coefficient of variation.

Table 2. PREFIT 20 m shuttle run (laps).

|  | ICC | 95% CI | SEM | MDC95 | CV% |
| --- | --- | --- | --- | --- | --- |
| Boys | 0.651 | 0.536 – 0.751 | 5.874 | 16.280 | 3.893 |
| Girls | 0.676 | 0.543 – 0.785 | 4.438 | 12.301 | 4.675 |
| 4 Year | 0.506 | 0.314 – 0.696 | 4.020 | 11.142 | 4.906 |
| 5 Year | 0.631 | 0.489 – 0.754 | 5.659 | 15.686 | 4.094 |
| 6 Year | 0.698 | 0.523 – 0.830 | 6.054 | 16.781 | 6.237 |
| Normal weight | 0.684 | 0.523 – 0.810 | 6.840 | 18.958 | 5.819 |
| Overweight | 0.696 | 0.481 – 0.849 | 4.588 | 12.716 | 6.357 |
| Obese | 0.595 | 0.322 – 0.819 | 4.784 | 13.261 | 6.847 |

ICC = Intraclass correlation; SEM = Standard Error of Measurement; MDC95 = Minimal Detectable Change at 95% confidence; CV% = percentage coefficient of variation.

Table 3. Handgrip strength (kg)

|  | ICC | 95% CI | SEM | MDC95 | CV% |
| --- | --- | --- | --- | --- | --- |
| Boys | 0.840 | 0.771 – 0.892 | 0.913 | 2.530 | 1.729 |
| Girls | 0.883 | 0.825 – 0.924 | 0.744 | 2.063 | 2.065 |
| 4 Year | 0.817 | 0.678 – 0.905 | 0.755 | 2.092 | 2.496 |
| 5 Year | 0.816 | 0.740 – 0.873 | 0.872 | 2.418 | 1.684 |
| 6 Year | 0.784 | 0.650 – 0.876 | 0.894 | 2.477 | 2.023 |
| Normal weight | 0.816 | 0.701 – 0.890 | 0.830 | 2.299 | 2.024 |
| Overweight | 0.779 | 0.617 – 0.885 | 1.048 | 2.904 | 2.832 |
| Obese | 0.841 | 0.702 – 0.922 | 0.7949 | 2.203 | 2.668 |

ICC = Intraclass correlation; SEM = Standard Error of Measurement; MDC95 = Minimal Detectable Change at 95% confidence; CV% = percentage coefficient of variation.

Table 4. Standing long jump (cm)

|  | ICC | 95% CI | SEM | MDC95 | CV% |
| --- | --- | --- | --- | --- | --- |
| Boys | 0.601 | 0.482 – 0.709 | 12.142 | 33.654 | 1.502 |
| Girls | 0.537 | 0.381 – 0.686 | 12.934 | 35.851 | 1.906 |
| 4 Year | 0.691 | 0.539 – 0.811 | 10.867 | 30.129 | 2.527 |
| 5 Year | 0.410 | 0.261 – 0.577 | 13.878 | 38.468 | 1.564 |
| 6 Year | 0.687 | 0.518 – 0.818 | 10.613 | 29.416 | 2.264 |
| Normal weight | 0.396 | 0.208 – 0.621 | 15.035 | 41.674 | 2.014 |
| Overweight | 0.488 | 0.137 – 0.851 | 11.385 | 31.556 | 2.338 |
| Obese | 0.772 | 0.586 – 0.890 | 9.497 | 26.323 | 3.339 |

ICC = Intraclass correlation; SEM = Standard Error of Measurement; MDC95 = Minimal Detectable Change at 95% confidence; CV% = percentage coefficient of variation.

Table 5. 4 × 10 m shuttle run (s)

|  | ICC | 95% CI | SEM | MDC95 | CV% |
| --- | --- | --- | --- | --- | --- |
| Boys | 0.777 | 0.691 – 0.844 | 0.932 | 2.582 | 0.721 |
| Girls | 0.780 | 0.663 – 0.864 | 0.831 | 2.302 | 0.793 |
| 4 Year | 0.808 | 0.673 – 0.896 | 0.824 | 2.283 | 0.933 |
| 5 Year | 0.679 | 0.564 – 0.776 | 0.999 | 2.770 | 0.726 |
| 6 Year | 0.761 | 0.617 – 0.863 | 0.695 | 1.926 | 0.943 |
| Normal weight | 0.756 | 0.626 – 0.852 | 0.821 | 2.276 | 0.924 |
| Overweight | 0.631 | 0.409 – 0.808 | 1.117 | 3.0953 | 1.233 |
| Obese | 0.693 | 0.481 – 0.846 | 0.883 | 2.4464 | 1.263 |

ICC = Intraclass correlation; SEM = Standard Error of Measurement; MDC95 = Minimal Detectable Change at 95% confidence; CV% = percentage coefficient of variation.

Table 6. One-leg stance (s)

|  | ICC | 95% CI | SEM | MDC95 | CV% |
| --- | --- | --- | --- | --- | --- |
| Boys | 0.518 | 0.353 – 0.680 | 6.636 | 18.393 | 3.469 |
| Girls | 0.457 | 0.276 – 0.650 | 6.742 | 18.687 | 4.197 |
| 4 Year | 0.236 | 0.062 – 0.590 | 3.952 | 10.954 | 2.561 |
| 5 Year | 0.330 | 0.163 – 0.556 | 7.165 | 19.860 | 3.539 |
| 6 Year | 0.523 | 0.294 – 0.743 | 8.105 | 22.466 | 6.964 |
| Normal weight | 0.447 | 0.178 – 0.751 | 8.041 | 22.287 | 5.387 |
| Overweight | 0.508 | 0.280 – 0.732 | 6.019 | 16.683 | 5.805 |
| Obese | 0.686 | 0.289 – 0.922 | 5.402 | 14.974 | 6.479 |

ICC = Intraclass correlation; SEM = Standard Error of Measurement; MDC95 = Minimal Detectable Change at 95% confidence; CV% = percentage coefficient of variation.

The results indicate that the physical assessments conducted demonstrate moderate to high reliability. The handgrip strength test exhibited substantial consistency between measurements, with an Intraclass Correlation Coefficient (ICC) of 0.86, accompanied by low values for Standard Error of Measurement (SEM) and Minimal Detectable Change at 95% confidence (MDC95), thereby indicating commendable accuracy. Likewise, the 4×10 m shuttle run test also demonstrated high reliability with an ICC of 0.78.In contrast, the PREFIT 20 m shuttle run test (ICC = 0.67) and the Standing long jump test (ICC = 0.58) revealed moderate reliability. The One-leg balance test had a lower ICC of 0.49, indicating low reliability and greater variability between measurements. Overall, the strength and agility tests emerged as the most reliable for this sample.
